# Encapsulation of Curcumin in Diblock Copolymer Micelles for Cancer Therapy

**DOI:** 10.1155/2015/824746

**Published:** 2015-02-22

**Authors:** Ali Mohammad Alizadeh, Majid Sadeghizadeh, Farhood Najafi, Sussan K. Ardestani, Vahid Erfani-Moghadam, Mahmood Khaniki, Arezou Rezaei, Mina Zamani, Saeed Khodayari, Hamid Khodayari, Mohammad Ali Mohagheghi

**Affiliations:** ^1^Cancer Research Center, Tehran University of Medical Sciences, Tehran 14197-33141, Iran; ^2^Department of Genetics, School of Biological Sciences, Tarbiat Modares University, Tehran 14115-137, Iran; ^3^Department of Resin and Additives, Institute for Color Science and Technology, Tehran 16765-654, Iran; ^4^Immunology Lab, Institute of Biochemistry and Biophysics, University of Tehran, Tehran 13145-1384, Iran; ^5^Department of Biotechnology, Faculty of Advanced Medical Technology, Golestan University of Medical Sciences, Gorgan 49175-553, Iran; ^6^Department of Pathology, School of Medicine, Tehran University of Medical Sciences, Tehran 14197-33141, Iran; ^7^School of Biological Science, Damghan University, Damghan 36716-41167, Iran; ^8^Cancer Model Research Center, Tehran University of Medical Sciences, Tehran 14197-33141, Iran

## Abstract

Application of nanoparticles has recently promising results for water insoluble agents like curcumin. In this study, we synthesized polymeric nanoparticle-curcumin (PNPC) and then showed its efficiency, drug loading, stability, and safety. Therapeutic effects of PNPC were also assessed on two cell lines and in an animal model of breast cancer. PNPC remarkably suppressed mammary and hepatocellular carcinoma cells proliferation (*P* < 0.05). Under the dosing procedure, PNPC was safe at 31.25 mg/kg and lower doses. Higher doses demonstrated minimal hepatocellular and renal toxicity in paraclinical and histopathological examinations. Tumor take rate in PNPC-treated group was 37.5% compared with 87.5% in control (*P* < 0.05). Average tumor size and weight were significantly lower in PNPC group than control (*P* < 0.05). PNPC increased proapoptotic Bax protein expression (*P* < 0.05). Antiapoptotic Bcl-2 protein expression, however, was lower in PNPC-treated animals than the control ones (*P* < 0.05). In addition, proliferative and angiogenic parameters were statistically decreased in PNPC-treated animals (*P* < 0.05). These results highlight the suppressing role for PNPC in* in vitro* and* in vivo* tumor growth models. Our findings provide credible evidence for superior biocompatibility of the polymeric nanocarrier in pharmacological arena together with an excellent tumor-suppressing response.

## 1. Introduction

Curcumin (1,7-bis (4-hydroxy-3-methoxy-phenyl)-1,6-heptadiene-3,5-dione; diferuloylmethane) is a natural, yellow, and lipid-soluble compound extracted from* Curcuma longa* plant with no discernible toxicity. Recent studies have shown that curcumin, either alone or in combination with other anticancer agents, has potent anticancer effects [1]. Curcumin is water insoluble and is eliminated mostly unchanged and partly altered in the gut. In preclinical and clinical studies, oral administration of curcumin yields low plasma and tissue concentrations. This may be due to low absorption, rapid metabolism and elimination, and limited systemic bioavailability [2]. To prevail over these weak points, different ways were tested such as piperine [3], liposomal curcumin [4, 5], and nanoparticle curcumin use [6, 7]. Application of nanoparticles has promising results for the poor water soluble hydrophobic agents like curcumin. Feasible preparation and use, size fitness, environmental sustainability, and shell viability are all important factors for a suitable carrier [8]. According to some studies, encapsulated form of curcumin improves its medical properties and solubility [9]. Previous studies showed that dendrosome, a diblock nanostructure made by oleic acid (OA) and polyethylene glycol (PEG, 400 Dalton) with anticancer and proapoptosis effects, is a suitable option for curcumin encapsulation [10–12]. In the present research, we used modified 2000 Dalton molecular weight monomethoxy-PEG (mPEG) for curcumin encapsulation. Recently our research group evaluated morphology and physical behavior of mPEG_2000_-OA [13]. Based on CMC measurement, this novel nanocarrier can be considered as an appropriate drug delivery system for curcumin delivery in cancer cells. A unique feature of mPEG_2000_-OA is the ease with which the structure of their monomers can be varied in order to provide suitable, inert drug porters for target cells. In this study, we exploited mPEG_2000_-OA for curcumin encapsulation as polymeric nanoparticle curcumin (PNPC) and investigated its efficiency, drug loading, and stability with more focus on clinical observations, hematological/blood chemistry tests, and histological examinations. In addition, PNPC's protective and therapeutic effects were also assessed in an animal model of breast cancer. Our results shed new light on PNPC potential biocompatibility in* in vitro* and* in vivo* biological systems.

## 2. Materials and Methods

### 2.1. Materials

Curcumin (>99%), doxorubicin hydrochloride, cyclophosphamide, ketamine, and xylazine were purchased from Sigma Aldrich Co. (St. Louis, MO, USA). Materials for the polymeric nanocarrier (OM2000) preparation including oleoyl chloride and methoxy polyethylene glycol 2000 (mPEG 2000) were got from Sigma-Aldrich Co. (St. Louis, MO, USA). Chloroform and triethyl amine were also purchased from Merck-Serono Company (Germany). The human hepatocellular (HuH-7), mouse mammary (4T1) carcinoma cell lines, and normal human fibroblast cells were procured from Pasteur Institute of Iran (Pasteur Institute, Tehran, Iran). Polyclonal mouse anti-rat/rabbit Bax, Bcl-2, and CD31 antibodies (DAKO Corporation, USA) and rabbit monoclonal antibody against Ki-67 (Thermo Fisher Scientific Inc., San Jose, CA) were purchased.

### 2.2. The Study Design

Studies were conducted in five series of experiments including (i) PNPC synthesis, (ii) PNPC stability and drug loading, (iii) PNPC effects on cell viability of mammary and hepatocellular carcinoma cells, (iv) PNPC acute and chronic toxicity, and (v) PNPC protective and therapeutic effects on animal model of breast cancer.

### 2.3. Animals

All animal studies have been conducted according to relevant national and international guidelines of the Weatherall report and Institutional Animal Care and Use Committee (IACUC) of Tehran University of Medical Sciences. Inbred female BALB/c mice (6–8 weeks old, purchased from Iran Pasteur Institute) were maintained under 12-hour dark and light cycle, with free access to food and water.

### 2.4. Polymeric Nanocarrier Synthesis

Polymeric nanoparticle (PNP, OM2000) was synthesized by esterification of oleoyl chloride (3.01 g, 0.01 mol) and methoxy polyethylene glycol 2000 (20 g, 0.01 mol) in the presence of triethyl amine (1.2 g, 0.012 mol) and chloroform as solvent at 25°C for 2 h (see Supplemental Figure  1 in Supplementary Materials available online at http://dx.doi.org/10.1155/2015/824746). Triethyl amine hydrochloride was filtered from organic phase. Chloroform was then evaporated and OM2000 dried in a 40°C vacuum oven for 4 h with 96% purity. Fourier transform infrared (FT-IR) spectroscopic measurements in the potassium bromide (KBr) pellets were done using a Perkin-Elmer spectrometer. The ^1^H NMR spectrums were carried out using Bruker 400 MHZ in DMSO-d_5_.

### 2.5. Critical Micelle Concentration Determination

Critical micelle concentration (CMC) of PNP was determined by detecting shifts in the pyrene fluorescence absorbance spectra [14]. In this regard, 3 mL of pyrene solution (6 × 10^−6^ M) in acetone was added to a glass test tube and evaporated to remove the solvent. Then, 5 mL of PNP (0.005 to 1 mg/mL) was conjugated with phosphate-buffered saline (PBS; 0.01 M, pH 7.4) added to the glass test tubes to reach to 6 × 10^−7^ M pyrene final concentration. The solutions were vortexed and conditioned at 37°C overnight. Fluorescence excitation spectra of pyrene (300–350 nm) were measured at an emission wavelength of 390 nm with slit widths of 2.5 and 5.0 nm (Perkin-Elmer Fluorimeter, USA) for excitation and emission, respectively. The fluorescence excitation shifts from 334 to 339 nm were used to determine CMC of the polymeric nanocarrier.

### 2.6. PNPC Physical Properties

Dynamic light scattering (DLS) and atomic force microscopy (AFM) were carried out to evaluate alterations in the zeta-potential, particle size, particle size distribution, shape, and polydispersity index (PdI) of the polymeric nanoparticle [14–16]. Nanoparticle zeta-potential and size distribution of PNPC (0.01 M PBS, pH 7.4) were analyzed by DLS (Zetasizer NanoZS, Malvern Instruments, UK) using an argon laser beam at 633 nm and 90° scattering angle. To determine PNPC shape, a drop of PNPC solution (0.05 mg/mL) was placed on freshly cleaved mica and was allowed to dry at room temperature. The sample was then mounted on a microscope scanner and imaged in semicontact mode with AFM (JPK Instruments Co., Germany).

### 2.7. PNPC Encapsulation Efficiency and Drug Loading

In this section, measurements were performed in triplicate. Different concentrations of curcumin (2 to 25 mg) in acetone solution were mixed with 100 mg nanocarrier in 3 mL of water and rotated with rotary evaporator till acetone evaporation. PNPC was filtered using 0.22 *μ*m syringe filter to remove nonencapsulated curcumin and then lyophilized. To disrupt micellar or vesicle structures, 10 mg lyophilized PNPC was dissolved in 1 mL methanol and vortexed with ultrasonic waves for 10 min to ensure encapsulated curcumin release. Curcumin concentration was then determined with spectrophotometer at 425 nm in comparison with methanol calibration curve for curcumin. Drug loading (DL) and encapsulation efficiency (EE) of curcumin/nanocarrier micelles were finally quantified utilizing the following equation [15, 16]:

(1) Drug  loading  content  % =weight  of  curcumin  in  micelles  weight  of  micelles×100,Encapsulation  efficiency  % =weight  of  micelled  curcumin  weight  of  feeding  curcumin  ×100.

### 2.8. PNPC Preparation

Curcumin (6 mg) was dissolved in acetone and added into nanocarrier solution (100 mg/3 mL water) and then mixed and rotated with rotary evaporator for acetone evaporation. PNPC was filtered using 0.22 *μ*m syringe filter to remove nonencapsulated curcumin. The complex was then lyophilized and used for next experiments [13].

### 2.9. Stability Assays

PNPC aqueous solution was kept at 4°C for 24 h and ten months at dark room temperature. Precipitation indicates PNPC instability, while clear solution confirms its stability [17]. Dissolved lyophilized samples in water were made by manual shaking without additional heating or sonication. PNPC size and distribution were compared with freshly prepared PNPC by DLS [18]. At the end of the first week, curcumin loaded content and size distribution of PNPC were compared with freshly prepared PNPC by spectroscopy and DLS, respectively.

### 2.10. MTT Assay

HuH-7, 4T1, and normal fibroblast cell lines were grown in Dulbecco's modified Eagle's medium (DMEM; GIBCO, USA) containing 10% fetal bovine serum (FBS; GIBCO, USA) at 37°C in humid atmosphere. Cell viability measured by MTT (3-[4,5-dimethylthiazol-2-yl] 2, 5-diphenyltetrazolium bromide) assay [19]. Curcumin stock solution (100 *μ*M) in DMEM was prepared from 10 mM curcumin in methanol. Methanol percentage in the final solution was lower than 0.4% v/v. Identical cell numbers (1 × 10^6^ cells) in 200 *μ*L DMEM containing 10% FBS were seeded in triplicate on 96-well plates and incubated overnight. Cells were subsequently treated with various concentrations (0, 10, 20, 30, and 40 *μ*M) of PNPC, PNP alone, curcumin, and doxorubicin (as positive control) for 24 h and 48 h. Afterwards, 20 *μ*L of MTT (5 mg/mL) was added to each well and incubated for an additional 4 h followed by adding 200 *μ*L of dimethyl sulfoxide. Relative cell viability was then determined using a 96-well plate reader (TECAN, Switzerland) at 540 nm.

### 2.11. Dosing Procedure

One hundred sixteen BALB/c mice were used to study the acute and chronic toxicity of PNPC [20]. In acute toxicity protocol, 2000 mg/kg of PNPC was given as starting dose. Then, doses of 2000, 1000, 500, 250, 125, 62.5, 31.25, and 15.63 mg/kg body weight of PNPC and PNP were intraperitoneally injected. Animals were euthanized after 24 h. In case of studying 24 h adverse reactions, the dose was adjusted based on the average toxicity and the last tolerated dose for the new group of mice. Organ damage, histopathological findings, abnormal hematological/blood chemical indices, reduced organ weight ratio, and body weight changes were amongst the toxicity signs.

Based on the acute toxicity results, chronic toxicity was carried out using doses of 125, 62.5, 31.25, and 15.63 mg/kg of PNPC and PNP for 7 consecutive days. The dose with no adverse reactions during 24 h was assigned as the survival dose. Survived animals were weighed on a daily basis and euthanized one week later. Hematology, blood chemistry, and histopathological tests were carried out. Vital organs including heart, liver, spleen, lung, brain, and kidney were excised and weighed.

### 2.12. Hematology, Blood Chemistry, and Histopathological Tests

Animals were decapitated under general anesthesia to evaluate hematology, clinical chemistry, and histopathology parameters. Blood samples were taken for clinical chemistry tests. Total leukocyte count (WBC), erythrocyte count (RBC), platelets (Plt), hemoglobin (Hgb), hematocrit (Hct), mean cell volume (MCV), mean corpuscular hemoglobin (MCH) and mean corpuscular hemoglobin concentration (MCHC), neutrophils, lymphocytes, eosinophils, and monocytes were measured using an animal blood counter (Celltac; Nihon Kohden, Tokyo, Japan). Plasma urea nitrogen (BUN), creatinine (Cr), sodium (Na), potassium (K), chloride (Cl), bicarbonate (HCO^3−^), calcium (Ca), magnesium (Mg), lactate (Lac), osmolarity (Osm), and glucose (Glu) were determined using CCX System (CCX* WB*; Nova Biomedical, USA). Plasma alkaline phosphatase (ALP), albumin (ALB), total bilirubin (T.Bil), direct bilirubin (D.Bil), gamma-glutamyl transpeptidase (GGT), alanine transaminase (ALT), and aspartate transaminase (AST) were also measured (Autoanalyser Model Biotecnica,* BT* 3500, Rome, Italy). In addition, liver, kidney, brain, lung, spleen, and heart tissue samples were fixed and preserved in 4% buffered formaldehyde for at least 24 h. Tissue blocks were prepared and evaluated for histopathological changes.

### 2.13. Tumor Transplantation

Spontaneous mouse mammary tumor (SMMT) was aseptically separated from the breast-cancer-bearing BALB/c mice, cut into pieces of less than 0.3 cm^3^, and subcutaneously transplanted into the animals' left flank under ketamine and xylazine (10 mg/kg, i.p.) anesthesia [21].

### 2.14. PNPC Protective Effects on an Animal Model of Breast Cancer

Sixteen mice were divided into two equal groups of nontreated (control) and treated for the study of PNPC protective effects on a typical animal model of breast cancer. PNPC was given for 24 days, from 3 days before to 21 days after tumor transplantation. Animal survival rate and tumor take rate were measured at the end of the study.

### 2.15. PNPC Therapeutic Effects on an Animal Model of Breast Cancer

Twenty-four mice were equally divided into negative control, positive control, and PNPC groups to study nanocurcumin therapeutic effects on mice breast cancer. PNPC was given for 24 days after tumor transplantation from day 14 up to day 38. In the positive control group, doxorubicin (5 mg/kg) and cyclophosphamide (2 mg/mouse) were intraperitoneally coadministered in three separate doses for three consecutive weeks. Normal saline was also given in the negative control group. Tumor volume was measured weekly by a digital vernier caliper (Mitutoyo, Japan) and reported according to the following formula [21]:

(2) $$\begin{array}{c} V=\frac{1}{6\left(\pi LWD\right)}, \end{array}$$

where *L* = length, *W* = width, and *D* = depth.

### 2.16. Histopathological Assay

The breast tumoral and adjacent nontumoral mucosal tissues were fixed in 10% formaldehyde, passaged, and embedded in paraffin. Paraffin blocks were then sectioned by 3–5 *μ*m thickness for hematoxylin and eosin (H&E) staining [22]. Slides were studied using OLYMPUS-BX51 microscope. Digital photos were taken with OLYMPUS-DP12 camera and graded by the Scarff-Bloom-Richardson Scale [23].

### 2.17. Immunohistochemistry Examinations

Immunohistochemistry was carried out on 3–5 *μ*m tissue sections taken from the formalin-fixed paraffin blocks using avidin-biotin immunoperoxidase method [24]. For tumor cells' apoptotic and angiogenic studies, sections were stained with polyclonal mouse anti-rat/rabbit Bax, Bcl-2, and CD31 antibodies (DAKO Corporation, USA) according to the manufacturer's instructions. Briefly, the paraffin sections were deparaffinized with xylene and rehydrated through a series of descending graded ethanol solutions. Slides kept into TBS-EDTA buffer and put into a microwave oven for 15 min at 90°C. Endogenous peroxidase activity was blocked by 0.3% H_2_O_2_ buffer incubation for 15 min. Biotinylated secondary antibody and avidin-biotin complex with horseradish peroxidase were applied followed by chromogen 3,3′-diaminobenzidine addition (Sigma Chemical).

To study tumor cell proliferative activity, sections were treated with 3% (v/v) H_2_O_2_ at room temperature, blocked with 10% (v/v) goat serum or rabbit serum (Nichirei, Tokyo, Japan), and incubated with a rabbit monoclonal antibody against Ki-67 (Thermo Fisher Scientific Inc., San Jose, CA). Sections were then incubated with biotinylated goat anti-rabbit IgG (Nichirei) and a solution of streptavidin-conjugated horseradish peroxidase (Nichirei) according to the manufacturer's recommendations.

Criteria used to evaluate Bax, Bcl-2, CD31, and Ki67 markers were based on the estimated proportion of positive cells and estimated average staining intensity of positive cells in cytoplasm (for Bax), membrane (for CD31), nucleus (for Ki67), and membrane, cytoplasm or nucleus (for Bcl-2). Semiquantitative score was adopted as follows [22, 24]:

 no staining: 0;
 faint/barely staining up to 1/3 of cells: 1,
 moderate staining in 1/3 to 1/2 of cells: 2,
 strong staining in more than 1/2 of cells: 3.

### 2.18. Statistical Analysis

Analysis of variance (ANOVA) and Tukey's post hoc tests were used for comparison between groups. Two-tailed Student's *t*-tests were used when comparing two groups. Differences in tumor incidence (percentage of animals with breast cancer) were analyzed by Fisher's exact probability test. Values were represented as mean ± SEM. *P* < 0.05 was considered to be statistically significant. Statistical analysis was done using SPSS statistical software version 14.0.

## 3. Results

### 3.1. The FT-IR Spectrum of the Polymeric Carrier

The FT-IR spectrum of the polymeric carrier showed stretching band of C–H aliphatic at 2889, 2947, and 2960 cm^−1^. C=O stretching vibration of ester bands could be seen at 1736 cm^−1^. C–H bending vibration of CH_2_ and C–H bending vibration of CH_3_ can be seen in 1467 and 1343 cm^−1^, respectively. C–O stretching vibration was at 1112 cm^−1^ as broad band (Supplemental Figure  2).

### 3.2. The ^1^H NMR Spectrum of the Polymeric Carrier

Supplemental Figure  3 shows ^1^H NMR spectrum of the polymeric carrier from 0 to 6.5 ppm in DMSO-d_5_. Saturated protons in fatty ester were at 0.8, 1.2, 1.5, 2, and 2.3 ppm. CH_2_ protons between unsaturated bonds in linoleate and linolenate were at 2.6 ppm. Residual DMSO-d_5_^1^H NMR signal was at 2.5 ppm (Supplemental Figure  3(a)). DMSO-d_5_ water was in 3.3 ppm as broad band. CH_3_ protons of mPEG were in 3.2 ppm. CH_2_ protons of mPEG ethylene oxide units were multipeaks in 3.5 ppm (Supplemental Figure  3(b)). CH_2_ protons of mPEG ethylene oxide connected to fatty acid chloride were at 4.1 and 4.2 ppm. C–H oleate, linoleate, and linolenate were in 5.3, 6.2, and 6.4 ppm, respectively (Supplemental Figure  3(c)).

### 3.3. Critical Micelle Concentration of Polymeric Nanocarrier

**Figure 1:**
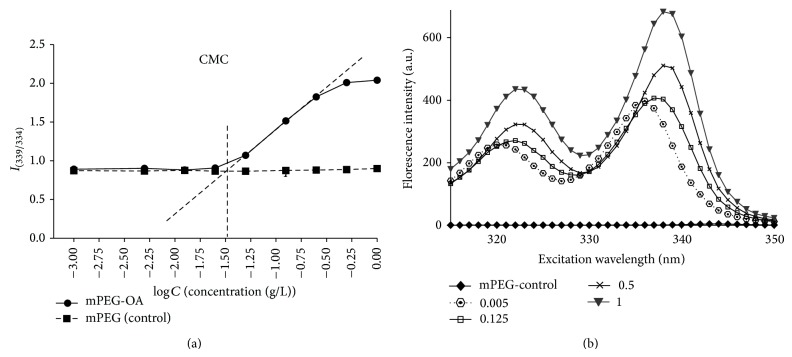
Determination of CMC of (a) pyrene excitation spectra shift and (b) four datasets from ten are displayed to simply show the below and above CMC concentration when micelles developed.* C*: the concentration of polymeric nanoparticle (PNP).

Concentration at crossover point in Figure 1 shows CMC of nanocarrier at 339/334 nm intensity ratio and nanocarrier concentration logarithm. As noted, CMC value is very low near 0.03 g/L. With increasing nanocarrier concentration, florescence intensity has significantly risen (Figure 1).

### 3.4. PNPC Physical Properties

**Figure 2:**
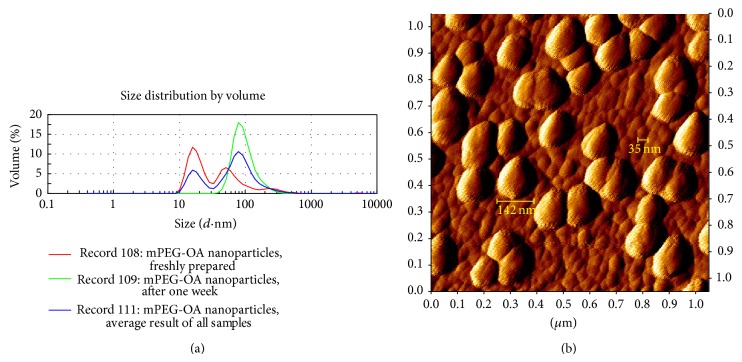
Morphology and particle size distribution of PNPC. (a) Red line shows average results of freshly prepared PNPC samples (0–25% curcumin encapsulation in PNP). It shows three particle sizes, 18.3 ± 5.3, 65.5 ± 30, and 283 ± 112 nm. Green line shows average results of PNPC samples after one week at 25°C (0–25% curcumin encapsulation in PNP). It shows one particle size of 99.4 ± 42.6 nm. Blue line shows average result of all samples with two sizes, 18.3 ± 5.3 (they seem to be micelles) and 99.4 ± 65 nm (they seem to be polymersomes). (b) Atomic force microscopy (AFM) results. AFM image of redissolved PNPC after freeze-drying (0.05 mg/mL) also showed two particle forms and sizes. Smaller particles (<40 nm) seem to be micelles and larger particles (>40 nm) seem to be polymersomes.

The size, morphology, and polydispersity of the nanoparticles were evaluated using dynamic light-scattering technique (DLS) and AFM methods (Figure 2). The results show that two forms of particles were produced in the process of synthesis, micelles and polymersomes with the average size of 18.33 ± 5.32 nm and 99.44 ± 65 nm, respectively. The freshly prepared PNP with different curcumin content (0–25%) were monodisperse (PdI = 0.332 ± 0.13), with three particle forms 53.5% micelles (18.33 ± 5.3 nm), 38.8% polymersomes (65.5 ± 30 nm), and 7.5% polymersomes (283.6 ± 112 nm). However, after one week of incubation at 25°C, the particle forms changed to be more monodisperse (PdI = 0.182 ± 0.072) with 100% polymersomes (99.44 ± 42.56 nm). The results of AFM analysis show that the shape of PNPC was in accordance with DLS analysis (Figure 2(b)). However, compared to DLS analysis, the AFM results show the larger size of PNPC which can be attributed to the expansion of spherical micelles or vesicles (polymersomes) in mica surface after drying its solution. Moreover, the AFM results show that in PNPC graph the* z*-dimensional (height) bar is smaller than* X*- and* Y*-dimensional bars which support this claim (Supplemental Figure  4). The PNPC is indeed stable in the presence of oleate in this nanoformulation. The negative zeta-potential was found to be −29.3 ± 5.2 mV at concentration of 0.05 mg/mL (slightly higher concentration than CMC point; 0.03 mg/mL). This low zeta-potential is at optimum range for stability of PNPC and explains the reason of developing more uniform size distribution (PdI = 0.182 ± 0.072) after one week at room temperature [25, 26].

### 3.5. PNPC Encapsulation Efficiency and Drug Loading

**Figure 3:**
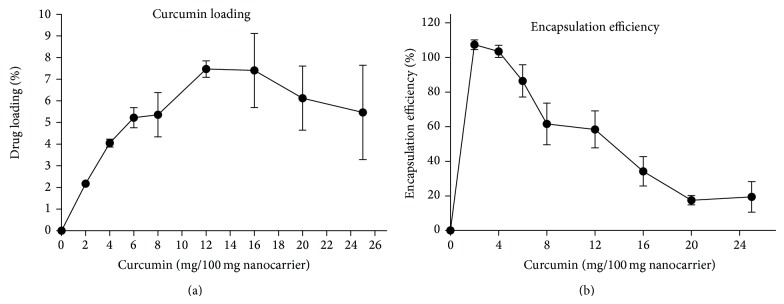
Encapsulation efficiency and drug loading of curcumin in PNPC. (a) Encapsulation efficiency (EE) and (b) drug loading (DL) of curcumin.

The average drug encapsulating efficiency and drug loading of PNPC were 64 and 5.97 ± 2.1% (w/w), respectively. The percentage of drug loading at different ratios of curcumin to 100 mg of nanocarrier was plotted in Figure 3. Curcumin did not precipitate at 6% loading after 10 months of incubation (Supplemental Figure  5).

### 3.6. PNPC Stability

**Figure 4:**
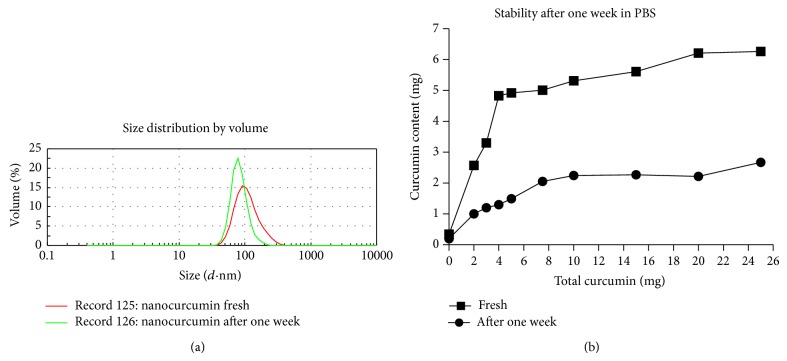
Stability test of curcumin/nanoparticle after one week at room temperature by DLS test.

PNPC was stable more than 300 days at 4°C, although loading curcumin higher than 6% into nanoparticles leads to some precipitation of curcumin (Supplemental Figure  7). Lyophilized PNPC samples in water dissolved by manual shaking without additional heating or sonication. Then, size and distribution of PNP were compared with freshly prepared PNPC by DLS (Figure 2(a)). After one week at 25°C, as mentioned, PNPC samples inclined to develop more uniform polymersomes nanoparticles (PdI = 0.182 ± 0.072). Furthermore, spectroscopy analysis showed that one-third of curcumin in curcumin/nanocarriers micelles (pH = 7.2) was conserved at room temperature after one week (Figure 4).

### 3.7. PNPC Effects on HuH-7 and 4T1 Cell Lines

**Figure 5:**
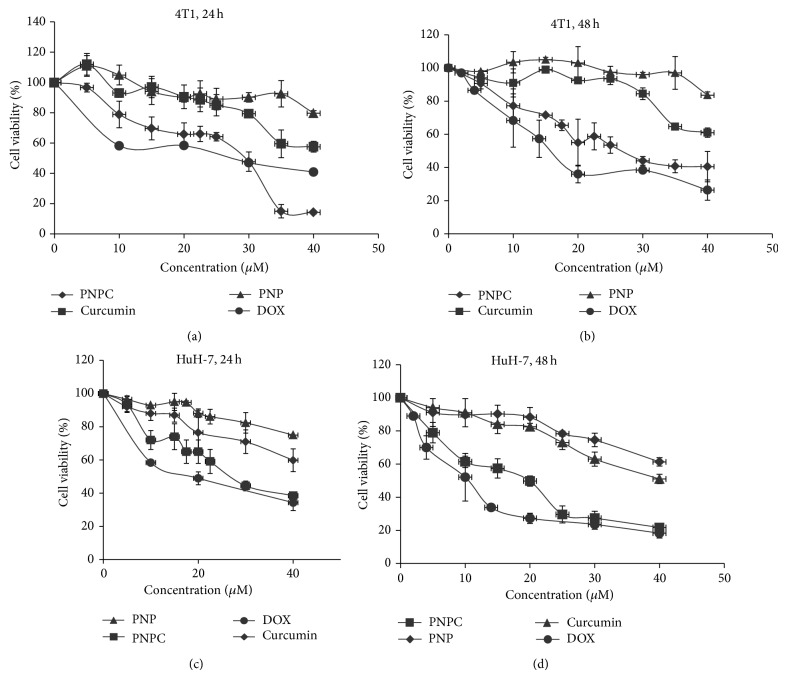
Cytotoxic effects of PNPC on mouse mammary (4T1) and human hepatocellular (HuH-7) carcinoma cells. Cells were treated with different concentrations of PNPC for 24 h (a) and 48 h (b) on 4T1 cell line and 24 h (c) and 48 h (d) on HuH-7 cell line. Data reported are mean ± SD; ^*^ *P* < 0.05 compared to curcumin; PNPC = the polymeric nanoparticle curcumin; PNP = polymeric nanoparticles.

PNPC significantly suppressed the proliferation of HuH-7 and 4T1 cancerous cells in a dose- and time-dependent manner in comparison with curcumin and PNP groups (*P* < 0.05). PNPC IC_50_ for 4T1 cells was 29 *μ*M within 24 h (Figure 5(a)), which relatively reduced to 24 *μ*M in 48 h (Figure 5(b)). In addition, PNPC IC_50_ for HuH-7 cells was 25 *μ*M within 24 h (Figure 5(c)), which relatively reduced to 19 *μ*M in 48 h (Figure 5(d)). We showed concentration about 48 and 40 *μ*M for IC_50_ of free curcumin for 24 and 48 h, respectively. Therefore, we showed that IC_50_ of the free curcumin is significantly higher than PNPC, and PNPC significantly suppressed cell growth compared to free curcumin (*P* < 0.05). In addition, no significant toxicity was observed for void mPEG-OA nanocarrier (PNP) even at concentrations above the 40 *μ*M. PNPC was not toxic to normal human fibroblastic cells (HFSF-PI3) [13], and this data is in accordance with other researcher results reviewed by Ravindran et al. [27]. PNPC showed nonsignificant changes in comparison with the positive control group. PNPC and doxorubicin had no effect on normal human fibroblasts cells (data not shown).

### 3.8. PNPC Toxicity

The main toxicity signs to PNPC and PNP in various doses are summarized in Tables 1 and 2 and Supplemental Tables  1–5. In acute toxicity groups, doses of 2000, 1000, 500, and 250 mg/kg of PNPC are associated with death or severe poisoning symptoms. Additionally, PNPC and PNP caused hematological, hepatocellular, and renal toxicity at the acute dose of 125 mg/kg (Table 1 and Supplemental Table  1). Higher doses provoked severe adverse reactions and death especially at 250 mg/kg PNPC.

In the chronic toxicity groups, doses up to 62.5 mg/kg PNPC brought about no death, but at 125 mg/kg PNPC, 2 out of 6 mice died. Weight loss, diarrhea, imbalance, and ascites were seen at 125 mg/kg of PNPC after one week of consecutive injections. Survived animals (31.25 mg/kg and lower) had no clinical differences compared with the control group. PNPC showed remarkable safety rates up to 31.25 mg/kg (Tables 1 and 2 and Supplemental Tables  1 and  2).

Hematological markers were measured by a complete blood count analysis. No inflammatory responses were seen in acute and chronic toxicity groups since total leukocyte counts remained within normal range (Tables 1 and 2 and Supplemental Table  1). In addition, significant increases in RBC, Hct, and Hgb were seen in acute 125 mg/kg PNPC and chronic 62.5 mg/kg PNPC groups compared to control animals (*P* < 0.05) (Table 1 and Supplemental Table  1). Na, K, and Cl were also statistically escalated, while HCO^3−^ drastically decreased in acute 125 mg/kg PNPC (Table 1) and chronic 62.5 mg/kg PNPC-treated mice compared with control groups (*P* < 0.05) (Table 2).

Plasma BUN and Cr levels were measured for kidney function assessment, while ALP, T.Bil, D.Bil, GGT, ALB, ALT, and AST were done for liver function evaluation. BUN and Cr were significantly higher in animals of acute 125 mg/kg PNPC (Tables 1 and 2) and chronic 62.5 mg/kg PNPC groups, compared to control animals (*P* < 0.05) (Table 2 and Supplemental Table  2). Furthermore, liver function tests showed drastic increases in AST, ALT, and ALP levels in acute 125 mg/kg PNPC group (Table 1 and Supplemental Table  1) and chronic 62.5 mg/kg PNPC group (Table 2 and Supplemental Table  1) compared to control mice (*P* < 0.05). On the other hand, albumin level significantly lowered at 62.5 mg/kg in chronic PNPC group compared to control animals (Table 2 and Supplemental Table  2). Liver and kidney weights were decreased but spleen weight increased in 125 mg/kg PNPC and PNP groups compared to controls groups (Table 2). It seems that the liver and the kidney were the target organs for the polymeric carrier toxicity in high doses.

In this study, we also injected 31.25 mg/kg PNPC for 7 consecutive days and animals were euthanized at the end of 2, 4, and 12 weeks. No adverse reactions observed in hematological, blood chemical, and histological examinations in this protocol (Supplemental Table  3).

We also compromised the toxicity of PNPC and PNP each other. In the acute toxicity, no significant difference was observed in hematological and blood chemical examinations in various doses of PNPC and PNP (Supplemental Table  4). But in the chronic groups, high BUN and AST levels and low albumin serum level were seen at 62.5 mg/kg PNP group compared to PNPC animals (Supplemental Table  5).

### 3.9. Histopathology Examinations

**Figure 6:**
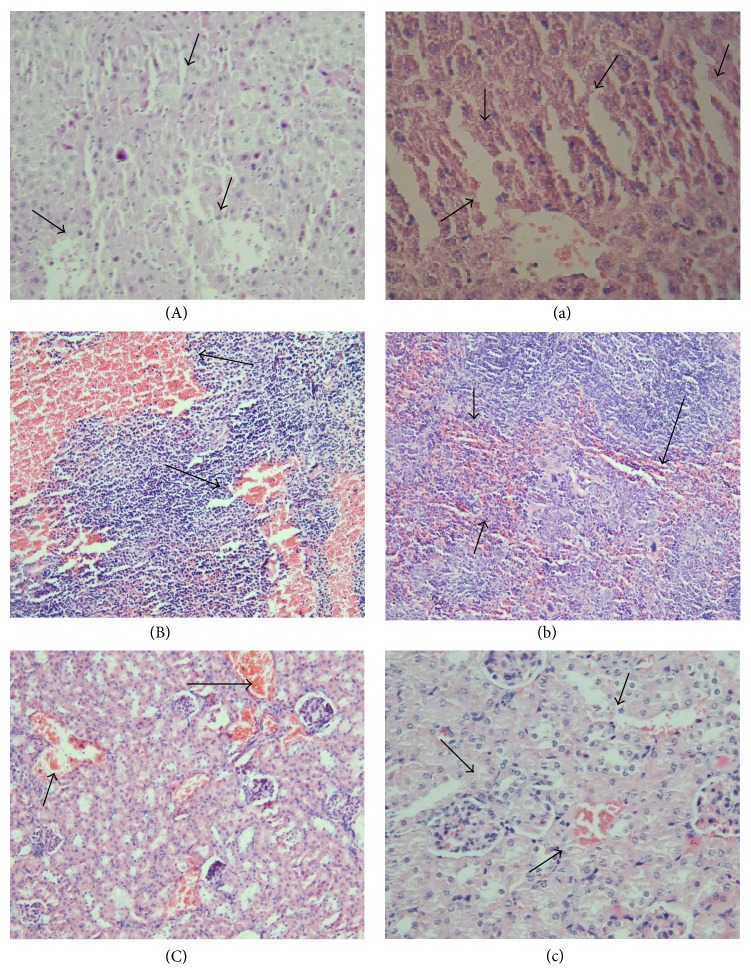
Light microscopic analysis of organs impressed from PNPC (125 mg/kg). Mild Kupffer cells hyperplasia and sinusoidal distention in favor of congestion were seen in the liver tissue [(A) (20x), (a) (40x)]. Moderate congestion, sinusoidal dilatation with preserved white lymphoid pulp was seen in the spleen tissue [(B) (20x), (b) (20x)]. The glomeruli were unremarkable but mild peritubular congestion and proteinaceous cast formations were noted in some kidney tubules [(C) (20x), (c) (40x)].

In order to obtain an accurate diagnosis of PNPC and PNP toxicity on microscopic levels, major organs were histopathologically evaluated. Compared with control, animals treated with 125 mg/kg PNPC and PNP developed slight abdominal ascites, kidney, liver, and spleen congestion after one-week consecutive injections. AST, ALT, ALP, and GGT drastic increases in these groups may be assumed as liver dysfunction. These changes were also congruent with elevated renal biomarkers. In the liver, mild Kupffer cells hyperplasia and sinusoidal distention in favor of congestion were seen (Figure 6(A)). In addition, we observed moderate congestion, sinusoidal dilatation with preserved white lymphoid pulp in the spleen tissue (Figure 6(B)). In the kidney, the glomeruli were unremarkable but mild peritubular congestion and proteinaceous cast formations were noted in some tubules (Figure 6(C)). In all of the histopathological examinations we observed minimal hepatic and renal toxicity. No histological abnormality was found in other major organs such as the brain, the heart, and the lung. Additionally, no significant pathologic changes were found in the major organs of animals treated with 31.25 mg/kg PNPC and lower doses compared with the control group, so 31.25 mg/kg dose was ruled out as the toxic dose.

### 3.10. PNPC Protective Effects on Animal Breast Cancer

In the protective study, PNPC was injected for 24 consecutive days, from 3 days before to 21 days after tumor transplantation. At the end of the study, the tumor take rate was at 37.5% in comparison with control group (87.5%; *P* < 0.05). Additionally, PNPC-treated mice had a greater survival rate of at least 20 weeks after tumor transplantation compared to the control animals who died by the end of the eighth week.

### 3.11. PNPC Therapeutic Effects on Animal Breast Cancer

**Figure 7:**
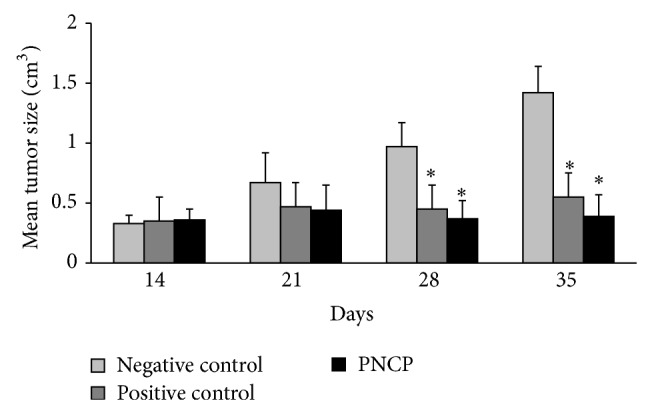
The polymeric nanoparticle curcumin effects on tumor size (cm^3^) in mouse mammary tumor. The positive control: “doxorubicin and cyclophosphamide were used as positive control.” Data reported are mean ± SD; ^*^ *P* < 0.05 compared to negative control; PNPC = polymeric nanoparticle curcumin.

PNPC was given for 24 days after tumor transplantation from day 14 up to day 38 to evaluate the therapeutic effects of PNPC on mice breast cancer. At the end of the third week, the tumor disappeared in 3 and the tumor volume decreased in 3 and increased in other two mice in PNPC group. Animals gained weight in PNPC group compared to the negative control. The average tumor volume and weight were significantly less than the negative control in the second and third weeks after PNPC treatment (*P* < 0.05). The mean final tumor volume reached approximately 390 mm^3^ and 1420 mm^3^ in PNPC-treated and control animals, respectively (Figure 7). The mean tumor volume and weight in the positive control group showed insignificant changes with PNPC group.

### 3.12. The Tumor Type and Characterization

Spontaneous mouse mammary tumor was invasive ductal carcinoma, malignant cells with hyperchromatic and polymorphism nuclei, and a low to moderate rate of mitosis. Tumor cell infiltration was observed in the surrounding tissues and nests of carcinoma cells with grade II/III based on Scarff-Bloom-Richardson Scale (Supplemental Figure  6).

### 3.13. Metastases

At the end of the study, all animals underwent routine surgery. There were no signs of metastasis in major organs of both PNPC-treated and control groups.

### 3.14. Immunohistochemistry Examinations

**Figure 8:**
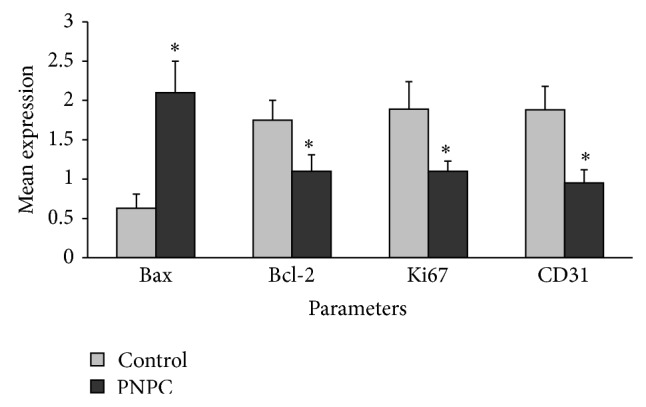
The polymeric nanoparticle curcumin effects on immunohistochemical markers in mouse mammary tumor. Data reported are mean ± SD; ^*^ *P* < 0.05 compared to control; PNPC = polymeric nanoparticle curcumin.

Immunohistochemistry examinations in PNPC-treated animals showed increased proapoptotic Bax protein expression in breast tumor in comparison with control (Figure 8 and Supplemental Figure  7A). Antiapoptotic Bcl-2 protein expression conceivably lowered after PNPC therapy (Figure 8 and Supplemental Figure  7B). In addition, treatment with PNPC caused a significant reduction in Bcl-2 activity and Bcl-2/Bax ratio compared to the control group (*P* < 0.05).

Angiogenesis also dramatically decreased in breast tumors of PNPC-treated animals. CD31 activity reduced in PNPC-treated mice tumors compared to control animals (Figure 8 and Supplemental Figure  7C). Moreover, most of the tumor cells represented high Ki67 marker in control group. The mean proliferative cell number in PNPC-treated mice was lower than the control group (*P* < 0.05) (Figure 8 and Supplemental Figure  7D).

## 4. Discussion

Our study showed that the polymeric nanocarrier is a negative, amphipathic, and biodegradable polymer suitable for drug delivery. They are new types of biocompatible polymeric chains taken from plant fatty acids suitable for curcumin bioavailability. Figure 1 shows that, with increasing nanocarrier concentration to more than CMC, the florescence intensity has significantly risen. This is due to the self-aggregation of micelle blocks and development of hydrophobic compartment inside the micelles and subsequent increase of pyrene florescence. Additionally, CMC low value indicates micelles thermodynamic stability which is enhanced after extreme* in vivo* dilution and may improve circulation time compared to surfactant micelles [14]. Another advantage of this nanocarrier is its small size which provokes passive targeting of the tumor tissues by increasing permeability and retention. Moreover, the stability of encapsulated curcumin in the polymeric nanoparticle is quite appropriate comparing with free curcumin which degrades to about 90% after 30 min [28]. Therefore, this nanocarrier conserves curcumin from decomposition and degradation and can be considered as an appropriate drug carrier for* in vitro* and* in vivo* experiments.

The major goal of our study was to develop polymeric nanoparticle curcumin as an applicable strategy in cancer treatment. Toxicity is a crucial factor for nanoparticle safety in nanomedicine arena. Understanding of various determinants of nanoparticle toxicity helps establishing suitable strategies for selecting appropriate compositions to develop biocompatible and efficacious polymeric carriers in nanomedicine requests. In our study, the polymeric nanoparticle curcumin significantly suppressed proliferation of human and mouse carcinoma cells* in vitro*. Based on hematological, blood chemical, and histopathological examinations, minimal hepatic and renal toxicity was seen with high PNPC doses. In addition,* in vivo* results showed that tumor incidence, weight, and size were significantly declined in PNPC-treated animals. PNPC also induced proapoptotic Bax protein expression and reduced antiapoptotic Bcl-2 protein levels relative to the control group. Moreover, proliferative and angiogenic markers were lowered in PNPC-treated animals. These findings point to the features of the polymeric carrier as a promising drug delivery system for cancer therapy.

In the present study, we showed that monotherapy with PNPC at high doses (250 mg/kg) and polymeric nanocarrier alone caused the animal acute death. Daily administration of PNPC and PNP at higher doses (125 mg/kg) for one week has also been life-threatening. Macroscopic changes such as ascites, weight loss, and imbalance together with other untoward sequels like splenomegaly were also seen in some animals. Histological examinations revealed macrophage infiltration and splenic hyperplasia. Sodium and K^+^ levels were significantly increased at high PNPC doses compared to the control animals. This indicates that fluid balance is somehow affected by PNPC administration and is in agreement with ascites and diarrhea observed in some animals at the same dose groups. RBC, Hct, and Hgb levels were drastically increased at 62.5 mg/kg and higher doses compared to the controls. Increased Cr level may be due to Cr release from damaged muscular cells [21]. We are not sure about the reversibility of PNPC and PNP induced blood chemistry and hematological changes since it is beyond the scope of the present study. Significant decrease in liver weight and albumin level together with increased ALT, AST, ALP, and GGT in PNPC- or PNP-treated animals points to the hepatic effects of high doses and may by induced by polymeric nanocarrier metabolism. Nevertheless, striking liver changes on the above mentioned parameters were seen in animals taking very high PNPC and PNP doses. These outcomes may have been primary or secondary effects from nanocarrier in some major organs such as liver, kidney, and spleen. Our results support this hypothesis that mPEG may trigger liver dysfunctions together with other organ toxicities [29, 30].

Our important finding is that the polymeric nanocarrier dramatically increased tumor-suppressing effects of curcumin both in cell culture and in a typical animal model of breast cancer. Our results also showed that tumor incidence, weight, and size were significantly declined in PNPC-treated group. Fairly complete tumor regression after 24 days is a dramatic finding with PNPC treatment observed in some animals. We also observed a decrease in tumor incidence as well as tumor size in both protective and PNPC-treated mice compared to the control group. Tumor incidence and size are indicators of proliferation and angiogenesis [31]. Changes in tumor growth characteristics observed in animals treated with PNPC suggest antiproliferative and antiangiogenic effects of PNPC. Lower tumor incidence and smaller tumor size may have been attributed to the direct effects of PNPC owing to its potential antiproliferative and antiangiogenic roles or its indirect strong oxidative response [32]. It should be noted that other pathways involved in the protective effects of PNPC are being pursued in our laboratory.

Apoptosis role in breast carcinogenesis has been extensively studied. It has been shown accordingly that resistance to apoptosis in premalignant breast epithelial cells can develop breast tumors [33]. In order to gain insight into the mechanisms involved in apoptosis induction mediated by PNPC in breast cancer, we studied its effects on the expression of Bcl-2 and Bax proteins under* in vivo* situations. Many genetic alterations of breast cancer are actually derived from an imbalance between pro- and antiapoptotic members of the Bcl-2 family [34]. It has generally been established that oncoprotein Bcl-2 duels with its counteracting twin, a protein known as Bax. Overexpression of Bax promotes cell death; conversely, Bcl-2 functions as a suppressor of apoptosis. A decrease in Bcl-2/Bax ratio has been considered as a reliable indicator of the overall propensity of a cell to undergo apoptosis [34]. In the present study, PNPC decreased Bcl-2/Bax ratio by suppressing Bcl-2 expression and Bax stimulation. This may be indicative of the Bcl-2 family role in apoptosis induction mediated by PNPC in mice breast cancer [35, 36]. These findings reveal a new therapeutic potential for PNPC in different tissues malignancies via apoptosis induction.

In addition, tumor growth noticeably depends on angiogenesis where simultaneous enhances in the tumor vasculature supply nutrients and oxygen to the growing neoplastic cells is required [37]. Growth and progression of breast tumor as well as growth of most of other cancers are angiogenesis-dependent processes. High angiogenic activity in the primary tumor seems to be well correlated with untoward sequels in patients suffering from breast cancer. Breast cancer is considered to be an angiogenic carcinoma due to high expression of proangiogenic factors [38]. We found* in vivo* anticarcinogenic properties of nanocurcumin as effective as* in vitro* antiproliferative and antiangiogenic effects of curcumin. These compounds presumably change the balance of pro-and antiangiogenic factors in tumor tissues and fix the effective delivery system of therapeutic drugs to tumor cells in a larger scale. Our results provide support for a potential therapeutic role of PNPC in breast cancer via their antiangiogenic and antiproliferative properties. In fact, it seems that the polymeric nanocarrier increases tumor cell access to curcumin and in turn, it causes tumor cells death.

In some previous reports, polymeric nanocarriers were used for curcumin tissue delivery. Bisht et al. (2007) synthesized a polymeric nanoparticle of curcumin (50–100 nm range) in an encapsulated formulation utilizing micellar aggregates of cross-linked and indiscriminate acrylic copolymers of N-isopropylacrylamide (NIPAAM) with N-vinyl-2-pyrrolidone (VP) [15]. An ideal drug delivery platform must be biodegradable, biocompatible, and free from incidental adverse effects. Bisht's nanocarriers are nondegradable and harmful to health due to NIPAAM, VP, and poly(ethylene glycol) monoacrylate monomers use. But, in our nanocarrier form, fatty acids with biodegradable and biocompatible properties were adopted to produce the least health harmful effects unless in very high doses. In addition, Anand et al. (2010) used PLGA-poly-ethylene glycol nanoparticles to deliver encapsulated curcumin with 97.5% efficiency but low drug loading [39]. Our data reveals for the first time that nanopolymeric compounds not only boost curcumin solubility and uptake in cell lines but also increase its toxicity on cancer cells. This issue with the biodegradable ability shows that polymeric carriers are unique host cell drug in animal models as per the following aspects.

- They are inexpensive, neutral, nontoxic, biodegradable, and easy to use.
- Pharmaceutical agents can create a weak structural combination helping their easy cellular separation.
- Although nanocarriers are biodegradable, they are stable and resistant not only in dry environments but also in fluids with low temperature.

## 5. Conclusion

In summary, we showed that PNPC is effective in suppressing tumor growth both* in vitro* and* in vivo*. Tumor growth in PNPC-treated mice was significantly suppressed and/or almost completely stopped at the end of the treatment. Our results also suggested PNPC appropriate dose (31.25 mg/kg/daily for 3 weeks). Lower doses effectiveness or fewer PNPC shots are currently being investigated in our laboratory. The present study provides persuasive evidence for polymeric nanocarrier superior biocompatibility in pharmacological arena which in turn can reduce anticancer drug side effects with excellent tumor-suppressing response.

## Supplementary Material

This content was submitted by the authors as supplemental material for an original article published in BioMed Research International. The content is presented as the author submitted it. All questions regarding the supplemental data should be directed to the corresponding author of the published article. It provides more experimental results on the encapsulation of curcumin in diblock copolymer micelles for cancer therapy.

## Figures and Tables

**Table 1:** Acute toxicity effects of PNPC on hematological and blood chemical indices in mice.

Parameters	Groups			
	Control	PNPC 31.25 mg/kg	PNPC 62.5 mg/kg	PNPC 125 mg/kg
Animal weight (g)	24.3 ± 2.2	25 ± 3.1	24.7 ± 2.5	23.2 ± 2.2
RBC (Millin/mm^3^)	6.08 ± 0.2	6.8 ± 0.8	7.2 ± 0.5	8.2 ± 0.4^*^
HCT (%)	38.4 ± 1.7	37.7 ± 1.2	38.5 ± 2.5	42.5 ± 2^*^
Hgb (g/dL)	10 ± 1.2	11.3 ± 1.8	11.7 ± 1.3	13.4 ± 1.6^*^
MCV (FL)	51 ± 0.7	50.1 ± 0.8	49.3 ± 1.5	49.1 ± 1.2
MCH (pg)	16.5 ± 0.2	16.4 ± 0.3	15.5 ± 0.4	15.3 ± 0.3
MCHC (mol/L)	32.3 ± 0.7	32.4 ± 0.5	32.2 ± 0.4	32.5 ± 0.3
Plt (1000/mm^3^)	567 ± 50	546 ± 91	570 ± 45	540 ± 38
WBC (1000/mm^3^)	6.7 ± 0.8	8.7 ± 2	7.9 ± 1.5	8.1 ± 2.2
Neutrophils (%)	35.5 ± 2	36.6 ± 12	38 ± 5	38.2 ± 6
Monocytes (%)	2.1 ± 3	1.2 ± 0.9	3.1 ± 1	2.1 ± 0.8
Lymphocytes (%)	53.5 ± 6.4	52 ± 9	57.2 ± 5	57 ± 4
Na (mM/L)	150.3 ± 2.9	146.5 ± 3.3	154.2 ± 2.1	157.1 ± 1.5^*^
K (mM/L)	5.2 ± 0.5	5.8 ± 1.3	6.1 ± 1.7	8.2 ± 2.2^*^
Cl (mM/L)	111 ± 4	117 ± 3	114 ± 2	117 ± 3^*^
HCO^3−^ (mM/L)	22.6 ± 3.2	17.3 ± 1.6	17.5 ± 2.2	16.3 ± 2.7^*^
Osm (mOsm/kg)	307 ± 4.7	307 ± 5.2	312.2 ± 7	320 ± 8^*^
Ca (mM/L)	0.72 ± 0.14	0.63 ± 0.1	0.65 ± 0.2	0.66 ± 0.3
Mg (mM/L)	0.29 ± 0.04	0.30 ± 0.03	0.25 ± 0.04	0.31 ± 0.03
Glu (mg/dL)	194 ± 33	204 ± 48	182 ± 30	176 ± 25
Lac (mM/L)	5.4 ± 1.2	5.2 ± 1.3	5.2 ± 2	5.7 ± 1.8
Urea (mg/dL)	21.2 ± 2.6	21.3 ± 3.6	26 ± 5	43 ± 7^*^
Cr (mg/dL)	0.48 ± 0.08	0.51 ± 0.07	0.55 ± 0.07	0.92 ± 0.12^*^
AST (U/L)	455 ± 45	538 ± 85	777 ± 80	1280 ± 152^*^
ALT (U/L)	68 ± 8.8	70.6 ± 8.7	86 ± 21	258 ± 86^*^
ALP (U/L)	763 ± 50	794 ± 67	825 ± 36	1187 ± 92^*^
GGT (U/L)	5.5 ± 0.5	—	—	6.2 ± 0.6
ALB (mg/dL)	3 ± 0.2	—	—	2.8 ± 0.4
T.BIL (mg/dL)	0.5 ± 0.03	—	—	0.47 ± 0.05
D.BIL (mg/dL)	0.35 ± 0.05	—	—	0.32 ± 0.04

Values are means ± SEM. ^*^ *P* < 0.05 compared to control. PNPC = the polymeric nanoparticle curcumin, RBC = red blood cell, HCT = hematocrit, MCV = mean corpuscular volume, MCH = mean corpuscular hemoglobin, MCHC = mean corpuscular hemoglobin concentration, WBC = white blood cells, Plt = platelets, Na = sodium, K = potassium, Cl = chloride, HCO^3−^ = bicarbonate, Osm = osmolarity, Ca = calcium, Mg = magnesium, Cr = creatinine, Lac = lactate, Glu = glucose, AST = aspartate transaminase, ALT = alanine transaminase, ALP = alkaline phosphatase, GGT = gamma-glutamyl transpeptidase, ALB = albumin, T.BIL = total bilirubin, and D.BIL = direct bilirubin.

**Table 2:** Chronic toxicity effects of PNPC on hematological and blood chemical indices and the organs weight percentage in mice.

Parameters	Groups			
	Control	PNPC 15.63 mg/kg	PNPC 31.25 mg/kg	PNPC 62.5 mg/kg
Animal weight (g)	24.3 ± 2.2	24 ± 3.2	23.3 ± 3.4	18.3 ± 2.7^*^
RBC (Millin/mm^3^)	6.08 ± 0.08	6.3 ± 0.07	6.9 ± 0.3	7.7 ± 0.4^*^
HCT (%)	38.4 ± 1.7	36.6 ± 2.1	37.4 ± 1.2	40.3 ± 3.2
Hgb (g/dL)	10 ± 1.2	12 ± 1.1	9.8 ± 0.9	12.3 ± 1
MCV (FL)	51 ± 0.7	52.8 ± 0.6	50.4 ± 2	49.5 ± 1.5
MCH (pg)	16.5 ± 0.2	17.3 ± 0.3	15.7 ± 0.9	15.6 ± 0.5
MCHC (mol/L)	32.3 ± 0.7	32.8 ± 0.4	32 ± 0.7	32.2 ± 0.6
Plt (1000/mm^3^)	567 ± 50	519 ± 75	539 ± 53	594 ± 41
WBC (1000/mm^3^)	6.7 ± 0.8	6.9 ± 1	6.5 ± 1.8	7.7 ± 2.1
Neutrophils (%)	32.5 ± 2	41 ± 8	38.5 ± 11	42 ± 9
Monocytes (%)	2.1 ± 3	4.4 ± 2.1	1.8 ± 1	3.8 ± 2.2
Lymphocytes (%)	66.5 ± 6.4	53 ± 11	60.8 ± 5.8	51 ± 13
Na (mM/L)	150.3 ± 2.9	149.7 ± 3.2	151.2 ± 3.8	157.4 ± 1.5^*^
K (mM/L)	5.2 ± 0.5	5.7 ± 1.6	4.9 ± 0.4	4.3 ± 0.8
Cl (mM/L)	111 ± 4	112.2 ± 2.1	112.7 ± 1.8	115.5 ± 2.3
HCO^3−^ (mM/L)	22.6 ± 3.2	16.7 ± 4.9	19.9 ± 3.2	18.9 ± 2.7
Osm (mOsm/kg)	307 ± 4.7	301.3 ± 12	306.9 ± 8.2	315.8 ± 7
Ca (mM/L)	0.72 ± 0.14	0.61 ± 0.14	0.74 ± 0.07	0.97 ± 0.6
Mg (mM/L)	0.29 ± 0.04	0.30 ± 0.02	0.32 ± 0.04	0.35 ± 0.03
Glu (mg/dL)	194 ± 33	174 ± 31	172 ± 40	212 ± 30
Lac (mM/L)	5.4 ± 1.2	4.8 ± 1	5.1 ± 1.1	4.3 ± 1.5
Urea (mg/dL)	21.2 ± 2.6	19.4 ± 3.2	20 ± 2.6	24.5 ± 3.8
Cr (mg/dL)	0.48 ± 0.08	0.42 ± 0.05	0.53 ± 0.03	0.98 ± 0.08^*^
AST (U/L)	455 ± 45	423 ± 64	503 ± 33	585 ± 36^*^
ALT (U/L)	68 ± 8.8	72.3 ± 5.9	76.3 ± 12	92.3 ± 5^*^
ALP (U/L)	763 ± 50	633 ± 83	666 ± 85	207 ± 23^*^
GGT (U/L)	5.5 ± 0.5	—	5.9 ± 0.8	6.4 ± 1
ALB (mg/dL)	3 ± 0.2	—	3.4 ± 0.6	2.3 ± 0.3^*^
T.BIL (mg/dL)	0.5 ± 0.03	—	0.47 ± 0.13	0.56 ± 0.1
D.BIL (mg/dL)	0.35 ± 0.05	—	0.38 ± 0.08	0.34 ± 0.05
% body weight				
Heart	0.52 ± 0.06			0.47 ± 0.04
Liver	6.3 ± 0.35			4.2 ± 0.26^*^
Spleen	0.43 ± 0.05			0.58 ± 0.07
Lung	0.73 ± 0.09			0.71 ± 0.07
Kidney	1.53 ± 0.15			1.46 ± 0.22
Brain	1.12 ± 0.11			1.19 ± 0.15

Values are means ± SEM. ^*^ *P* < 0.05 compared to control. PNPC = the polymeric nanoparticle curcumin, RBC = red blood cell, HCT = hematocrit, MCV = mean corpuscular volume, MCH = mean corpuscular hemoglobin, MCHC = mean corpuscular hemoglobin concentration, WBC = white blood cells, Plt = platelets, Na = sodium, K = potassium, Cl = chloride, HCO^3−^ = bicarbonate, Osm = osmolarity, Ca = calcium, Mg = magnesium, Cr = creatinine, Lac = lactate, Glu = glucose, AST = aspartate transaminase, ALT = alanine transaminase, ALP = alkaline phosphatase, GGT = gamma-glutamyl transpeptidase, ALB = albumin, T.BIL = total bilirubin, and D.BIL = direct bilirubin.

## References

[1] Bayet-Robert M., Morvan D. (2013). Metabolomics reveals metabolic targets and biphasic responses in breast cancer cells treated by curcumin alone and in association with docetaxel. *PLoS ONE*.

[2] Anand P., Kunnumakkara A. B., Newman R. A., Aggarwal B. B. (2007). Bioavailability of curcumin: problems and promises. *Molecular Pharmaceutics*.

[3] Kakarala M., Brenner D. E., Korkaya H. (2010). Targeting breast stem cells with the cancer preventive compounds curcumin and piperine. *Breast Cancer Research and Treatment*.

[4] Dhule S. S., Penfornis P., Frazier T. (2012). Curcumin-loaded *γ*-cyclodextrin liposomal nanoparticles as delivery vehicles for osteosarcoma. *Nanomedicine: Nanotechnology, Biology, and Medicine*.

[5] Ghalandarlaki N., Alizadeh A. M., Ashkani-Esfahani S. (2014). Nanotechnology-applied curcumin for different diseases therapy. *BioMed Research International*.

[6] Shutava T. G., Balkundi S. S., Vangala P. (2009). Layer-by-layer-coated gelatin nanoparticles as a vehicle for delivery of natural polyphenols. *ACS Nano*.

[7] Le Droumaguet B., Nicolas J., Brambilla D. (2012). Versatile and efficient targeting using a single nanoparticulate platform: application to cancer and alzheimer's disease. *ACS Nano*.

[8] Ledley F. D. (1995). Nonviral gene therapy: the promise of genes as pharmaceutical products. *Human Gene Therapy*.

[9] Suwannateep N., Wanichwecharungruang S., Haag S. F. (2012). Encapsulated curcumin results in prolonged curcumin activity *in vitro* and radical scavenging activity *ex vivo* on skin after UVB-irradiation. *European Journal of Pharmaceutics and Biopharmaceutics*.

[10] Babaei E., Sadeghizadeh M., Hassan Z. M., Feizi M. A. H., Najafi F., Hashemi S. M. (2012). Dendrosomal curcumin significantly suppresses cancer cell proliferation *in vitro* and *in vivo*. *International Immunopharmacology*.

[11] Khaniki M., Azizian S., Alizadeh A. M., Hemmati H., Emamipour N., Mohagheghi M. A. (2013). The antiproliferative and anticancerogenic effects of nano-curcumin in rat colon cancer. *Tehran University Medical Journal*.

[12] Sarbolouki M. N., Alizadeh A. M., Khaniki M., Azizian S., Mohaghgheg M. A. (2012). Protective effect of dendrosomal curcumin combination on colon cancer in rat. *Tehran University Medical Journal*.

[13] Mirgani M. T., Isacchi B., Sadeghizadeh M. (2014). Dendrosomal curcumin nanoformulation downregulates pluripotency genes via miR-145 activation in U87MG glioblastoma cells. *International Journal of Nanomedicine*.

[14] Sahu A., Bora U., Kasoju N., Goswami P. (2008). Synthesis of novel biodegradable and self-assembling methoxy poly(ethylene glycol)-palmitate nanocarrier for curcumin delivery to cancer cells. *Acta Biomaterialia*.

[15] Bisht S., Feldmann G., Soni S., Ravi R., Karikar C., Maitra A. (2007). Polymeric nanoparticle-encapsulated curcumin (“nanocurcumin”): a novel strategy for human cancer therapy. *Journal of Nanobiotechnology*.

[16] Ma Z., Haddadi A., Molavi O., Lavasanifar A., Lai R., Samuel J. (2008). Micelles of poly(ethylene oxide)-b-poly(*ε*-caprolactone) as vehicles for the solubilization, stabilization, and controlled delivery of curcumin. *Journal of Biomedical Materials Research Part A*.

[17] Gou M., Men K., Shi H. (2011). Curcumin-loaded biodegradable polymeric micelles for colon cancer therapy in vitro and in vivo. *Nanoscale*.

[18] Song L., Shen Y., Hou J., Lei L., Guo S., Qian C. (2011). Polymeric micelles for parenteral delivery of curcumin: preparation, characterization and *in vitro* evaluation. *Colloids and Surfaces A: Physicochemical and Engineering Aspects*.

[19] Mosmann T. (1983). Rapid colorimetric assay for cellular growth and survival: application to proliferation and cytotoxicity assays. *Journal of Immunological Methods*.

[20] Sharma R. A., Gescher A. J., Steward W. P. (2005). Curcumin: the story so far. *European Journal of Cancer*.

[21] Mohsenikia M., Alizadeh A. M., Khodayari S. (2013). The protective and therapeutic effects of alpha-solanine on mice breast cancer. *European Journal of Pharmacology*.

[22] Alizadeh A. M., Faghihi M., Khori V. (2012). Oxytocin protects cardiomyocytes from apoptosis induced by ischemia-reperfusion in rat heart: role of mitochondrial ATP-dependent potassium channel and permeability transition pore. *Peptides*.

[23] Achard J.-L., van Praagh I., Feillel V. (2002). Scarff-Bloom-Richardson (SBR) grading: a pleiotropic marker of chemosensitivity in invasive ductal breast carcinomas treated by neoadjuvant chemotherapy. *International Journal of Oncology*.

[24] Alizadeh A. M., Khaniki M., Azizian S., Mohaghgheghi M. A., Sadeghizadeh M., Najafi F. (2012). Chemoprevention of azoxymethane-initiated colon cancer in rat by using a novel polymeric nanocarrier-curcumin. *European Journal of Pharmacology*.

[25] Vila A., Sánchez A., Tobío M., Calvo P., Alonso M. J. (2002). Design of biodegradable particles for protein delivery. *Journal of Controlled Release*.

[26] Müller R. H., Jacobs C., Kayser O. (2001). Nanosuspensions as particulate drug formulations in therapy: rationale for development and what we can expect for the future. *Advanced Drug Delivery Reviews*.

[27] Ravindran J., Prasad S., Aggarwal B. B. (2009). Curcumin and cancer cells: how many ways can curry kill tumor cells selectively?. *AAPS Journal*.

[28] Wang Y.-J., Pan M.-H., Cheng A.-L. (1997). Stability of curcumin in buffer solutions and characterization of its degradation products. *Journal of Pharmaceutical and Biomedical Analysis*.

[29] Heyes J., Hall K., Tailor V., Lenz R., MacLachlan I. (2006). Synthesis and characterization of novel poly(ethylene glycol)-lipid conjugates suitable for use in drug delivery. *Journal of Controlled Release*.

[30] Wei X., Gong C., Gou M. (2009). Biodegradable poly(*ε*-caprolactone)-poly(ethylene glycol) copolymers as drug delivery system. *International Journal of Pharmaceutics*.

[31] Maeda K., Chung Y.-S., Takatsuka S. (1995). Tumour angiogenesis and tumour cell proliferation as prognostic indicators in gastric carcinoma. *British Journal of Cancer*.

[32] Zingg J.-M., Hasan S. T., Meydani M. (2013). Molecular mechanisms of hypolipidemic effects of curcumin. *BioFactors*.

[33] Mommers E. C. M., van Diest P. J., Leonhart A. M., Meijer C. J. L. M., Baak J. P. A. (1999). Balance of cell proliferation and apoptosis in breast carcinogenesis. *Breast Cancer Research and Treatment*.

[34] Baell J. B., Huang D. C. S. (2002). Prospects for targeting the Bcl-2 family of proteins to develop novel cytotoxic drugs. *Biochemical Pharmacology*.

[35] Karunagaran D., Rashmi R., Santhosh Kumar T. R. (2005). Induction of apoptosis by curcumin and its implications for cancer therapy. *Current Cancer Drug Targets*.

[36] Anto R. J., Mukhopadhyay A., Denning K., Aggarwal B. B. (2002). Curcumin (diferuloylmethane) induces apoptosis through activation of caspase-8, BID cleavage and cytochrome c release: its suppression by ectopic expression of Bcl-2 and Bcl-xl. *Carcinogenesis*.

[37] Folkman J. (1990). What is the evidence that tumors are angiogenesis dependent?. *Journal of the National Cancer Institute*.

[38] Fox S. B., Generali D. G., Harris A. L. (2007). Breast tumour angiogenesis. *Breast Cancer Research*.

[39] Anand P., Nair H. B., Sung B. (2010). Design of curcumin-loaded PLGA nanoparticles formulation with enhanced cellular uptake, and increased bioactivity *in vitro* and superior bioavailability *in vivo*. *Biochemical Pharmacology*.

