# Supplementary material for: Encapsulation of Curcumin in Diblock Copolymer Micelles for Cancer Therapy
Source: Biomed Res Int. 2015 Feb 22;2015:824746. doi: 10.1155/2015/824746 (PMC4352453; doi:10.1155/2015/824746)

Supplemental Data

Supplemental Table 1. Acute toxicity effects of alone PNP on hematological and blood chemical indices in mice

Groups / Parameters	Control	PNP 31.25 mg/kg	PNP 62.5 mg/kg	PNP 125 mg/kg
Animal Weight (g)	24.3 ± 2.2	22 ± 3.3	23.4 ± 2.6	23.4 ± 2.7
RBC (Millin/mm^3)	6.08 ± 0.2	6.5 ± 0.9	7.6 ± 0.6	*8.8 ± 0.6
HCT (%)	36.4 ± 1.5	36.7 ± 1.8	38.6 ± 2.7	*40.1 ± 2
Hgb (g/dl)	10 ± 1.2	11.6 ± 1.9	12.8 ± 1.9	*14.6 ± 1.6
MCV (FL)	51 ± 0.7	51.1 ± 0.9	49.9 ± 1	52.1 ± 1.2
MCH (pg)	16.5 ± 0.2	16.5 ± 0.4	16.5 ± 0.6	16.3 ± 0.5
MCHC (mol/l)	32.3 ± 0.7	32.6 ± 0.6	32.6 ± 0.8	32.4 ± 0.4
Plt (1000/mm^3)	567 ± 50	560 ± 71	570 ± 46	578 ± 66
WBC (1000/mm^3)	6.7 ± 0.8	7.5 ± 2.1	7.8 ± 1.8	7.9 ± 2.4
Neutrophils (%)	35.5 ± 2.3	36.7 ± 5	39 ± 6	41 ± 7
Monocytes (%)	2.1 ± 0.4	2.2 ± 0.4	3.2 ± 1	3.1 ± 0.8
Lymphocytes (%)	56.5 ± 6.7	55 ± 7	56 ± 6	54 ± 6
Na (mM/l)	150.3 ± 2.9	152 ± 3	154 ± 2	*169.1 ± 5
K (mM/l)	5.2 ± 0.5	5.3 ± 1.2	6.3 ± 1.8	*7.7 ± 2
Cl (mM/l)	111 ± 4	115 ± 4	114 ± 5	*119 ± 4
HCO^{3-} (mM/l)	22.6 ± 3.2	21.2 ± 2	19.6 ± 2	*15.3 ± 2.3
Osm (mOsm/kg)	307 ± 4.7	308 ± 5	310.2 ± 5	*325 ± 6
Ca (mM/l)	0.72 ± 0.14	0.66 ± 0.2	0.75 ± 0.3	0.88 ± 0.3
Mg (mM/l)	0.29 ± 0.04	0.31 ± 0.02	0.34 ± 0.05	0.36 ± 0.04
Glu (mg/dl)	194 ± 33	190 ± 56	210 ± 39	214 ± 45
Lac (mM/l)	5.4 ± 1.2	5.3 ± 1.4	5.5 ± 2.3	5.8 ± 1.9
Urea (mg/dl)	21.2 ± 2.6	24.3 ± 2.6	27 ± 6	*48 ± 9
Cr (mg/dl)	0.48 ± 0.08	0.52 ± 0.2	0.56 ± 0.2	*1.06 ± 0.2
AST (U/l)	455 ± 45	438 ± 80	529 ± 66	*1500 ± 165
ALT (U/l)	68 ± 8.8	73.6 ± 9.7	116 ± 55	*377 ± 87
ALP (U/l)	763 ± 50	788 ± 55	843 ± 46	*1200 ± 76
GGT (U/l)	5.5 ± 0.5	5.4 ± 0.3	5.7 ± 0.4	*8.2 ± 0.9
ALB (mg/dl)	3 ± 0.2	3.2 ± 0.3	2.7 ± 0.5	3.5 ± 0.5
T.BIL (mg/dl)	0.5 ± 0.03	0.45 ± 0.13	0.55 ± 0.1	0.59 ± 0.09
D.BIL (mg/dl)	0.35 ± 0.05	0.39 ± 0.09	0.35 ± 0.1	0.3 ± 0.09

Values are means ± SEM. *$P<0.05$ compared to control. PNP = The polymeric nanoparticle, RBC= Red blood cell, HCT= hematocrit, MCV = Mean Corpuscular Volume, MCH= Mean Corpuscular Hemoglobin, MCHC= Mean Corpuscular Hemoglobin Concentration, WBC= White blood cells, Plt = Platelets, Na = Sodium, K = Potassium, Cl = Chloride, HCO^{3-} = Bicarbonate, Osm = osmolarity, Ca = Calcium, Mg =Magnesium, Cr = Creatinine, Lac = Lactate, Glu = Glucose, AST = Aspartate transaminase, ALT = Alanine transaminase, ALP = Alkaline phosphatase, GGT = Gamma-glutamyl transpeptidase, ALB = Albumin, T.BIL = Total bilirubin, D.BIL = Direct Bilirubin.

Supplemental Table 2. Chronic toxicity effects of PNP on hematological and blood chemical indices in mice

Groups / Parameters	Control	PNP 15.63 mg/kg 1 W	PNP 31.25 mg/kg 1 W	PNP 62.5 mg/kg 1 W
Animal Weight (g)	24.3 ± 2.2	23 ± 2.2	23.8 ± 2.5	*18.2 ± 3.1
RBC (Millin/mm^3)	6.08 ± 0.08	6.5 ± 0.1	6.7 ± 0.4	*8.5 ± 0.5
HCT (%)	36.4 ± 1.7	36.8 ± 2.6	37.5 ± 2.2	42.2 ± 3.3
Hgb (g/dl)	11 ± 1.2	11.5 ± 1.4	10.8 ± 1.4	14.5 ± 2
MCV (FL)	51 ± 0.7	52.1 ± 0.7	51.2 ± 1.5	52.5 ± 1.3
MCH (pg)	16.5 ± 0.2	16.6 ± 0.6	16.7 ± 0.8	16.6 ± 0.7
MCHC (mol/l)	32.3 ± 0.7	32.5 ± 0.5	32.3 ± 0.7	32.7 ± 0.8
Plt (1000/mm^3)	567 ± 50	579 ± 86	555 ± 59	584 ± 87
WBC (1000/mm^3)	6.7 ± 0.8	7.4 ± 1.1	7.2 ± 1.9	7.3 ± 1.8
Neutrophils (%)	32.5 ± 2	38 ± 9	35.3 ± 7	39 ± 6
Monocytes (%)	2.1 ± 3	3.3 ± 2.1	2.8 ± 1.3	2.9 ± 1.2
Lymphocytes (%)	66.5 ± 6.4	57 ± 12	63.6 ± 7.2	58 ± 11
Na (mM/l)	150.3 ± 2.9	149.7 ± 3.2	151.2 ± 3.8	*164.2 ± 5.5
K (mM/l)	5.2 ± 0.5	5.7 ± 1.6	4.9 ± 0.4	*7.4 ± 1.6
Cl (mM/l)	111 ± 4	113.6 ± 3.1	113.7 ± 2.8	*119.5 ± 2.5
HCO^{3-} (mM/l)	22.6 ± 3.2	16.7 ± 4.9	19.9 ± 3.2	*16.9 ± 2.7
Osm (mOsm/kg)	307 ± 4.7	303.4 ± 10	310.9 ± 9.3	317.8 ± 9
Ca (mM/l)	0.72 ± 0.14	0.63 ± 0.24	0.71 ± 0.27	0.87 ± 0.09
Mg (mM/l)	0.29 ± 0.04	0.31 ± 0.05	0.33 ± 0.06	0.37 ± 0.03
Glu (mg/dl)	194 ± 33	214 ± 35	182 ± 45	89 ± 32
Lac (mM/l)	5.4 ± 1.2	4.9 ± 1.2	5.4 ± 1.3	4.7 ± 1.8
Urea (mg/dl)	21.2 ± 2.6	21.4 ± 3.3	25 ± 2.8	*38.5 ± 3
Cr (mg/dl)	0.48 ± 0.08	0.47 ± 0.06	0.57 ± 0.05	*1.20 ± 0.1
AST (U/l)	455 ± 45	460 ± 69	513 ± 43	*789 ± 55
ALT (U/l)	68 ± 8.8	74.3 ± 7.9	79.3 ± 15	*102± 10
ALP (U/l)	763 ± 50	640 ± 73	789 ± 75	*207 ± 23
GGT (U/l)	5.5 ± 0.5	5.7 ± 0.4	5.8 ± 0.6	*8.5 ± 0.9
ALB (mg/dl)	3 ± 0.2	3.4 ± 0.4	3.5 ± 0.5	*1.5 ± 0.4
T.BIL (mg/dl)	0.5 ± 0.03	0.5 ± 0.07	0.55 ± 0.1	0.56 ± 0.09
D.BIL (mg/dl)	0.35 ± 0.05	0.36 ± 0.06	0.33 ± 0.04	0.37 ± 0.07

Values are means ± SEM. *$P<0.05$ compared to control. PNP = The polymeric nanoparticle, RBC= Red blood cell, HCT= hematocrit, MCV = Mean Corpuscular Volume, MCH= Mean Corpuscular Hemoglobin, MCHC= Mean Corpuscular Hemoglobin Concentration, WBC= White blood cells, Plt = Platelets, Na = Sodium, K = Potassium, Cl = Chloride, HCO^{3-} = Bicarbonate, Osm = osmolarity, Ca = Calcium, Mg =Magnesium, Cr = Creatinine, Lac = Lactate, Glu = Glucose, AST = Aspartate transaminase, ALT = Alanine transaminase, ALP = Alkaline phosphatase, GGT = Gamma-glutamyl transpeptidase, ALB = Albumin, T.BIL = Total bilirubin, D.BIL = Direct Bilirubin.

Supplemental Table 3. Chronic toxicity effects of PNPC (31.25 mg/kg) on hematological and blood chemical indices in mice

Groups / Parameters	Control	PNPC 1 W	PNPC 2 W	PNPC 4 W	PNPC 12 W
Animal Weight (g)	24.3 ± 2.2	26.3 ± 3.4	28.1 ± 3	30.2 ± 3.1	32.3 ± 3.2
RBC (Millin/mm^3)	6.08 ± 0.08	6.9 ± 0.3	6.3 ± 0.5	6.8 ± 0.8	7.2 ± 0.9
HCT (%)	38.4 ± 1.7	37.4 ± 1.2	39.4 ± 3.1	39.1 ± 2.2	39.4 ± 2.7
Hgb (g/dl)	10 ± 1.2	9.8 ± 0.9	11.7 ± 1.6	12.1 ± 2.5	11.5 ± 2.6
MCV (FL)	51 ± 0.7	50.4 ± 2	49.5 ± 0.7	49 ± 1.1	50 ± 0.9
MCH (pg)	16.5 ± 0.2	15.7 ± 0.9	16.2 ± 0.2	16.8 ± 0.5	16.1 ± 0.3
MCHC (mol/l)	32.3 ± 0.7	32 ± 0.7	32.6 ± 0.2	32.3 ± 0.7	32.3 ± 0.7
Plt (1000/mm^3)	567 ± 50	539 ± 53	525 ± 47	580 ± 70	570 ± 58
WBC (1000/mm^3)	6.7 ± 0.8	6.5 ± 1.8	8.4 ± 2.1	7.1 ± 0.9	7.3 ± 0.9
Neutrophils (%)	32.5 ± 2	38.5 ± 11	39 ± 4.1	37.5 ± 4	35.5 ± 0.9
Monocytes (%)	2.1 ± 3	1.8 ± 1	3.5 ± 1.5	3.1 ± 1.2	3.7 ± 1.2
Lymphocytes (%)	66.5 ± 6.4	60.8 ± 5.8	58.2 ± 7.1	60.5 ± 5.4	61.5 ± 5.7
Na (mM/l)	150.3 ± 2.9	151.2 ± 3.8	153.5 ± 2.2	153.3 ± 2.8	154.1 ± 3.3
K (mM/l)	5.2 ± 0.5	4.9 ± 0.4	6.1 ± 2.0	6.2 ± 0.8	6.5 ± 0.7
Cl (mM/l)	111 ± 4	112.7 ± 1.8	110.5 ± 3.2	115 ± 6	115 ± 5
HCO^{3-} (mM/l)	22.6 ± 3.2	19.9 ± 3.2	22.8 ± 2.5	25.5 ± 4.2	26.6 ± 5.4
Osm(mOsm/kg)	307 ± 4.7	306.9 ± 8.2	309 ± 6	312 ± 5.7	313 ± 5.2
Ca (mM/l)	0.72 ± 0.14	0.74 ± 0.07	0.81 ± 0.2	0.82 ± 0.2	0.85 ± 0.15
Mg (mM/l)	0.29 ± 0.04	0.32 ± 0.04	0.29 ± 0.02	0.33 ± 0.07	0.35 ± 0.6
Glu (mg/dl)	194 ± 33	172 ± 40	208 ± 27	214 ± 35	204 ± 30
Lac (mM/l)	5.4 ± 1.2	5.1 ± 1.1	6.2 ± 2.1	5.2 ± 1.1	5.0 ± 2.2
Urea (mg/dl)	21.2 ± 2.6	20 ± 2.6	25.7 ± 3.5	25.2 ± 3.6	24.4 ± 3.4
Cr (mg/dl)	0.48 ± 0.08	0.53 ± 0.03	0.58 ± 0.15	0.62 ± 0.09	0.61 ± 0.07
AST (U/l)	455 ± 45	503 ± 33	568 ± 61	495 ± 55	555 ± 60
ALT (U/l)	68 ± 8.8	76.3 ± 12	77 ± 18	84 ± 17	78 ± 23
ALP (U/l)	763 ± 50	666 ± 85	872 ± 98	863 ± 90	843 ± 75
GGT (U/l)	5.5 ± 0.5	-	-	5.2 ± 0.6	5.6 ± 0.5
ALB (mg/dl)	3 ± 0.2	-	-	3.1 ± 0.3	3.2 ± 0.4
T.BIL (mg/dl)	0.5 ± 0.03	-	-	0.48 ± 0.04	0.52 ± 0.03
D.BIL (mg/dl)	0.35 ± 0.05	-	-	0.36 ± 0.04	0.37 ± 0.06

Values are means ± SEM.*P<0.05 compared to control. PNPC = The polymeric nanoparticle curcumin, RBC= Red blood cell, HCT= hematocrit, MCV = Mean Corpuscular Volume, MCH= Mean Corpuscular Hemoglobin, MCHC= Mean Corpuscular Hemoglobin Concentration, WBC= White blood cells, Plt = Platelets, Na = Sodium, K = Potassium, Cl = Chloride, HCO^{3-} = Bicarbonate, Osm = osmolarity, Ca = Calcium, Mg =Magnesium, Cr = Creatinine, Lac = Lactate, Glu = Glucose, AST = Aspartate transaminase, ALT = Alanine transaminase, ALP = Alkaline phosphatase, GGT = Gamma-glutamyl transpeptidase, ALB = Albumin, T.BIL = Total bilirubin, D.BIL = Direct Bilirubin.

Supplemental Table 4. Acute toxicity effects of high dose of PNPC and PNP on hematological and blood chemical indices in mice

Groups / Parameters	PNP 62.5 mg/kg	PNP 125 mg/kg	PNPC 62.5 mg/kg	PNPC 125 mg/kg
Animal Weight (g)	23.4 ± 2.6	23.4 ± 2.7	24.7 ± 2.5	23.2 ± 2.2
RBC (Millin/mm^3)	7.6 ± 0.6	8.8 ± 0.6	7.2 ± 0.5	8.2 ± 0.4
HCT (%)	38.6 ± 2.7	40.1 ± 2	38.5 ± 2.5	42.5 ± 2
Hgb (g/dl)	12.8 ± 1.9	14.6 ± 1.6	11.7 ± 1.3	13.4 ± 1.6
MCV (FL)	49.9 ± 1	52.1 ± 1.2	49.3 ± 1.5	49.1 ± 1.2
MCH (pg)	16.5 ± 0.6	16.3 ± 0.5	15.5 ± 0.4	15.3 ± 0.3
MCHC (mol/l)	32.6 ± 0.8	32.4 ± 0.4	32.2 ± 0.4	32.5 ± 0.3
Plt (1000/mm^3)	570 ± 46	578 ± 66	570 ± 45	540 ± 38
WBC (1000/mm^3)	7.8 ± 1.8	7.9 ± 2.4	7.9 ±1.5	8.1 ± 2.2
Neutrophils (%)	39 ± 6	41 ± 7	38 ± 5	38.2 ± 6
Monocytes (%)	3.2 ± 1	3.1 ± 0.8	3.1 ± 1	2.1 ± 0.8
Lymphocytes (%)	56 ± 6	54 ± 6	57.2 ± 5	57 ± 4
Na (mM/l)	154 ± 2	169.1 ± 5	154.2 ± 2.1	157.1 ± 1.5
K (mM/l)	6.3 ± 1.8	7.7 ± 2	6.1 ± 1.7	8.2 ± 2.2
Cl (mM/l)	114 ± 5	119 ± 4	114 ± 2	117 ± 3
HCO^{3-} (mM/l)	19.6 ± 2	15.3 ± 2.3	17.5 ± 2.2	16.3 ± 2.7
Osm (mOsm/kg)	310.2 ± 5	325 ± 6	312.2 ± 7	320 ± 8
Ca (mM/l)	0.75 ± 0.3	0.88 ± 0.3	0.65 ± 0.2	0.66 ± 0.3
Mg (mM/l)	0.34 ± 0.05	0.36 ± 0.04	0.25 ± 0.04	0.31 ± 0.03
Glu (mg/dl)	210 ± 39	214 ± 45	182 ± 30	176 ± 25
Lac (mM/l)	5.5 ± 2.3	5.8 ± 1.9	5.2 ± 2	5.7 ± 1.8
Urea (mg/dl)	27 ± 6	48 ± 9	26 ± 5	43 ± 7
Cr (mg/dl)	0.56 ± 0.2	1.06 ± 0.2	0.55 ± 0.07	0.92 ± 0.12
AST (U/l)	529 ± 66	1500 ± 165	777 ± 80	1280 ± 152
ALT (U/l)	116 ± 55	377 ± 87	86 ± 21	258 ± 86
ALP (U/l)	843 ± 46	1200 ± 76	825 ± 36	1187 ± 92
GGT (U/l)	5.7 ± 0.4	8.2 ± 0.9	-	6.2 ± 0.6
ALB (mg/dl)	2.7 ± 0.5	3.5 ± 0.5	-	2.8 ± 0.4
T.BIL (mg/dl)	0.55 ± 0.1	0.59 ± 0.09	-	0.47 ± 0.05
D.BIL (mg/dl)	0.35 ± 0.1	0.3 ± 0.09	-	0.32 ± 0.04

Values are means ± SEM. $^{*}P<0.05$ compared to PNCP. PNP = The polymeric nanoparticle, PNPC = The polymeric nanoparticle curcumin, RBC= Red blood cell, HCT= hematocrit, MCV = Mean Corpuscular Volume, MCH= Mean Corpuscular Hemoglobin, MCHC= Mean Corpuscular Hemoglobin Concentration, WBC= White blood cells, Plt = Platelets, Na = Sodium, K = Potassium, Cl = Chloride, HCO^{3-} = Bicarbonate, Osm = osmolarity, Ca = Calcium, Mg =Magnesium, Cr = Creatinine, Lac = Lactate, Glu = Glucose, AST = Aspartate transaminase, ALT = Alanine transaminase, ALP = Alkaline phosphatase, GGT = Gamma-glutamyl transpeptidase, ALB = Albumin, T.BIL = Total bilirubin, D.BIL = Direct Bilirubin.

Supplemental Table 5. Chronic toxicity effects of high dose of PNPC and PNP on hematological and blood chemical indices and the organs weight percentage in mice

Groups / Parameters	PNP 31.25 mg/kg	PNP 62.5 mg/kg	PNPC 31.25 mg/kg	PNPC 62.5 mg/kg
Animal Weight (g)	23.8 ± 2.5	18.2 ± 3.1	23.3 ± 3.4	18.3 ± 2.7
RBC (Millin/mm^3)	6.7 ± 0.4	8.5 ± 0.5	6.9 ± 0.3	7.7 ± 0.4
HCT (%)	37.5 ± 2.2	42.2 ± 3.3	37.4 ± 1.2	40.3 ± 3.2
Hgb (g/dl)	10.8 ± 1.4	14.5 ± 2	9.8 ± 0.9	12.3 ± 1
MCV (FL)	51.2 ± 1.5	52.5 ± 1.3	50.4 ± 2	49.5 ± 1.5
MCH (pg)	16.7 ± 0.8	16.6 ± 0.7	15.7 ± 0.9	15.6 ± 0.5
MCHC (mol/l)	32.3 ± 0.7	32.7 ± 0.8	32 ± 0.7	32.2 ± 0.6
Plt (1000/mm^3)	555 ± 59	584 ± 87	539 ± 53	594 ± 41
WBC (1000/mm^3)	7.2 ± 1.9	7.3 ± 1.8	6.5 ± 1.8	7.7 ± 2.1
Neutrophils (%)	35.3 ± 7	39 ± 6	38.5 ± 11	42 ± 9
Monocytes (%)	2.8 ± 1.3	2.9 ± 1.2	1.8 ± 1	3.8 ± 2.2
Lymphocytes (%)	63.6 ± 7.2	58 ± 11	60.8 ± 5.8	51 ± 13
Na (mM/l)	151.2 ± 3.8	164.2 ± 5.5	151.2 ± 3.8	157.4 ± 1.5
K (mM/l)	4.9 ± 0.4	5.4 ± 1.6	4.9 ± 0.4	4.3 ± 0.8
Cl (mM/l)	113.7 ± 2.8	119.5 ± 2.5	112.7 ± 1.8	115.5 ± 2.3
HCO^{3-} (mM/l)	19.9 ± 3.2	16.9 ± 2.7	19.9 ± 3.2	18.9 ± 2.7
Osm (mOsm/kg)	310.9 ± 9.3	317.8 ± 9	306.9 ± 8.2	315.8 ± 7
Ca (mM/l)	0.71 ± 0.27	0.87 ± 0.09	0.74 ± 0.07	0.97 ± 0.6
Mg (mM/l)	0.33 ± 0.06	0.37 ± 0.03	0.32 ± 0.04	0.35 ± 0.03
Glu (mg/dl)	182 ± 45	89 ± 32	172 ± 40	212 ± 30
Lac (mM/l)	5.4 ± 1.3	4.7 ± 1.8	5.1 ± 1.1	4.3 ± 1.5
Urea (mg/dl)	25 ± 2.8	*38.5 ± 3	20 ± 2.6	24.5 ± 3.8
Cr (mg/dl)	0.57 ± 0.05	1.20 ± 0.1	0.53 ± 0.03	0.98 ± 0.08
AST (U/l)	513 ± 43	*789 ± 55	503 ± 33	585 ± 36
ALT (U/l)	79.3 ± 15	102± 10	76.3 ± 12	92.3 ± 5
ALP (U/l)	789 ± 75	211 ± 33	666 ± 85	207 ± 23
GGT (U/l)	5.8 ± 0.6	8.5 ± 0.9	5.9 ± 0.8	6.4 ± 1
ALB (mg/dl)	3.5 ± 0.5	*1.5 ± 0.4	3.4 ± 0.6	2.3 ± 0.3
T.BIL (mg/dl)	0.55 ± 0.1	0.56 ± 0.09	0.47 ± 0.13	0.56 ± 0.1
D.BIL (mg/dl)	0.33 ± 0.04	0.37 ± 0.07	0.38 ± 0.08	0.34 ± 0.05

Values are means ± SEM. *$P<0.05$ compared to PNPC. PNP = The polymeric nanoparticle, PNPC = The polymeric nanoparticle curcumin, RBC= Red blood cell, HCT= hematocrit, MCV = Mean Corpuscular Volume, MCH= Mean Corpuscular Hemoglobin, MCHC= Mean Corpuscular Hemoglobin Concentration, WBC= White blood cells, Plt = Platelets, Na = Sodium, K = Potassium, Cl = Chloride, HCO^{3-} = Bicarbonate, Osm = osmolarity, Ca = Calcium, Mg =Magnesium, Cr = Creatinine, Lac = Lactate, Glu = Glucose, AST = Aspartate transaminase, ALT = Alanine transaminase, ALP = Alkaline phosphatase, GGT = Gamma-glutamyl transpeptidase, ALB = Albumin, T.BIL = Total bilirubin, D.BIL = Direct Bilirubin.

Supplemental data Fig. 1.

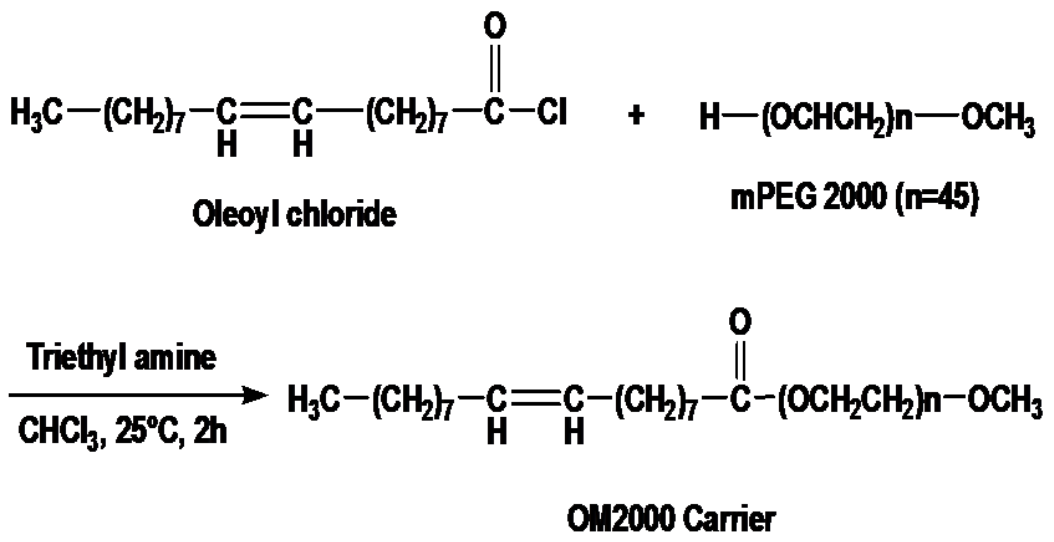

Supplemental data Fig. 2.

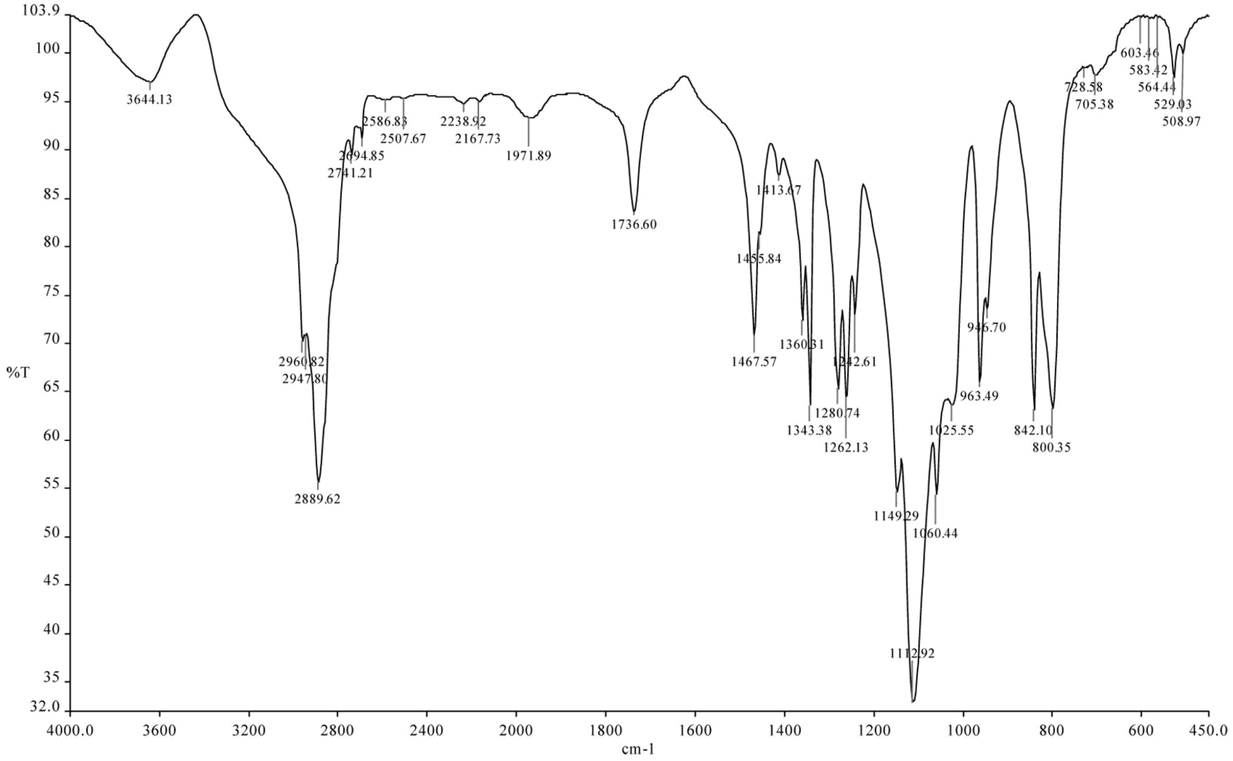

Supplemental data Fig. 3A.

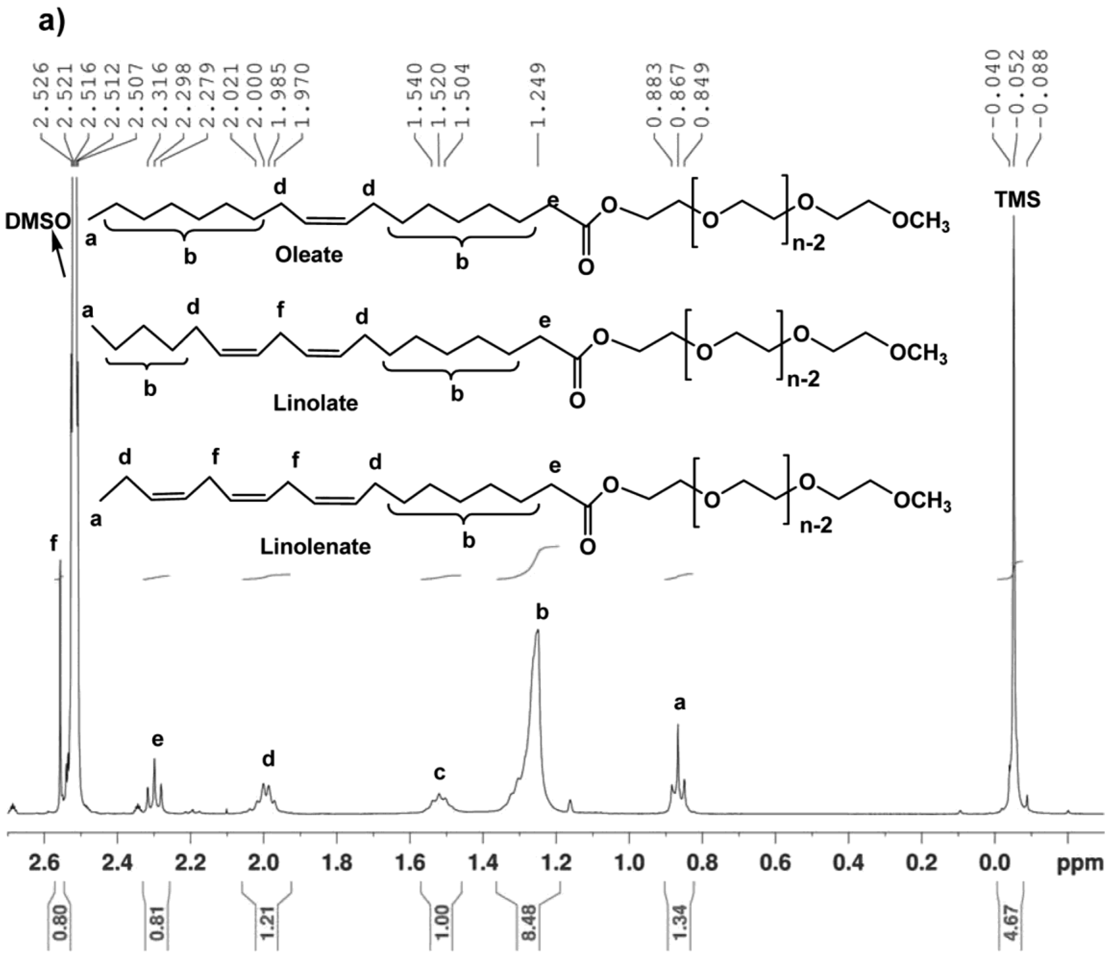

Supplemental data Fig. 3B.

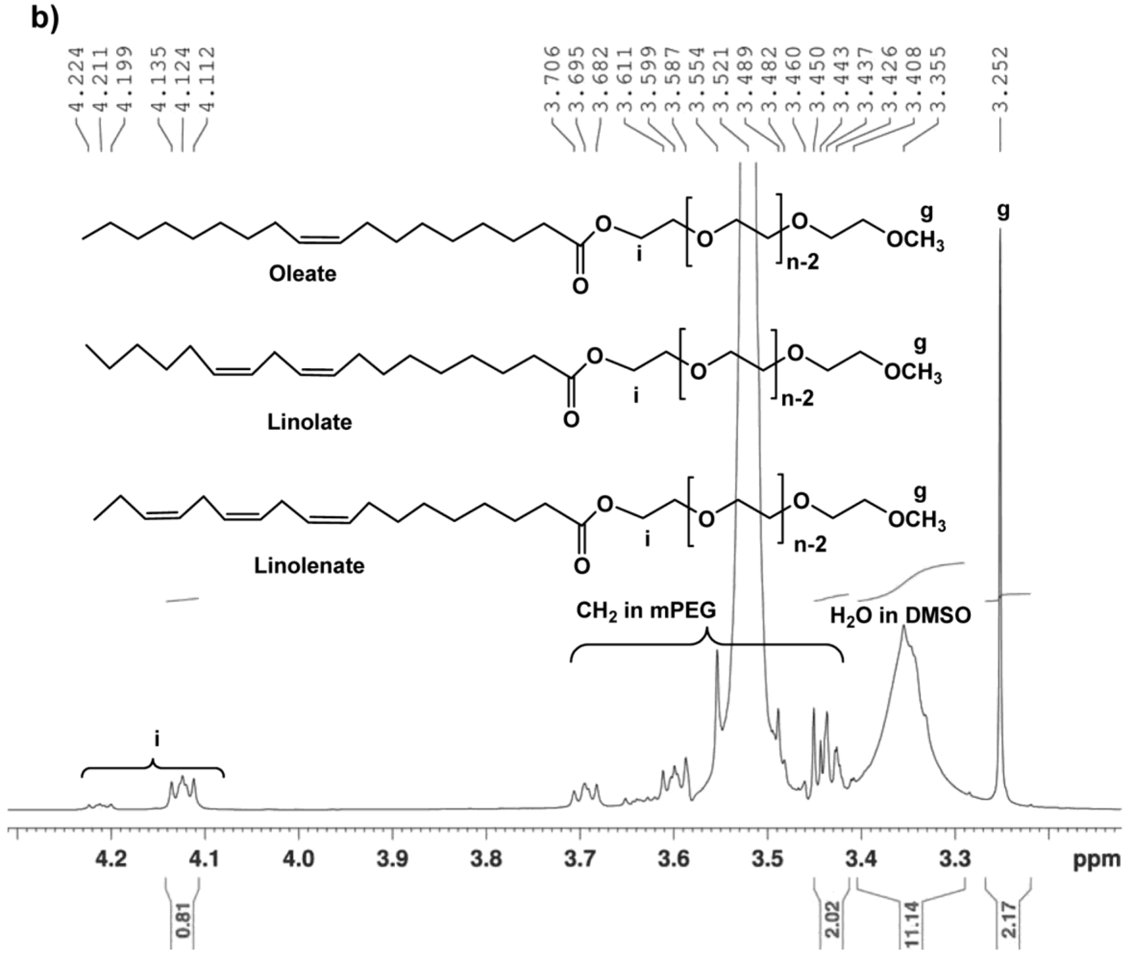

Supplemental data Fig. 3C.

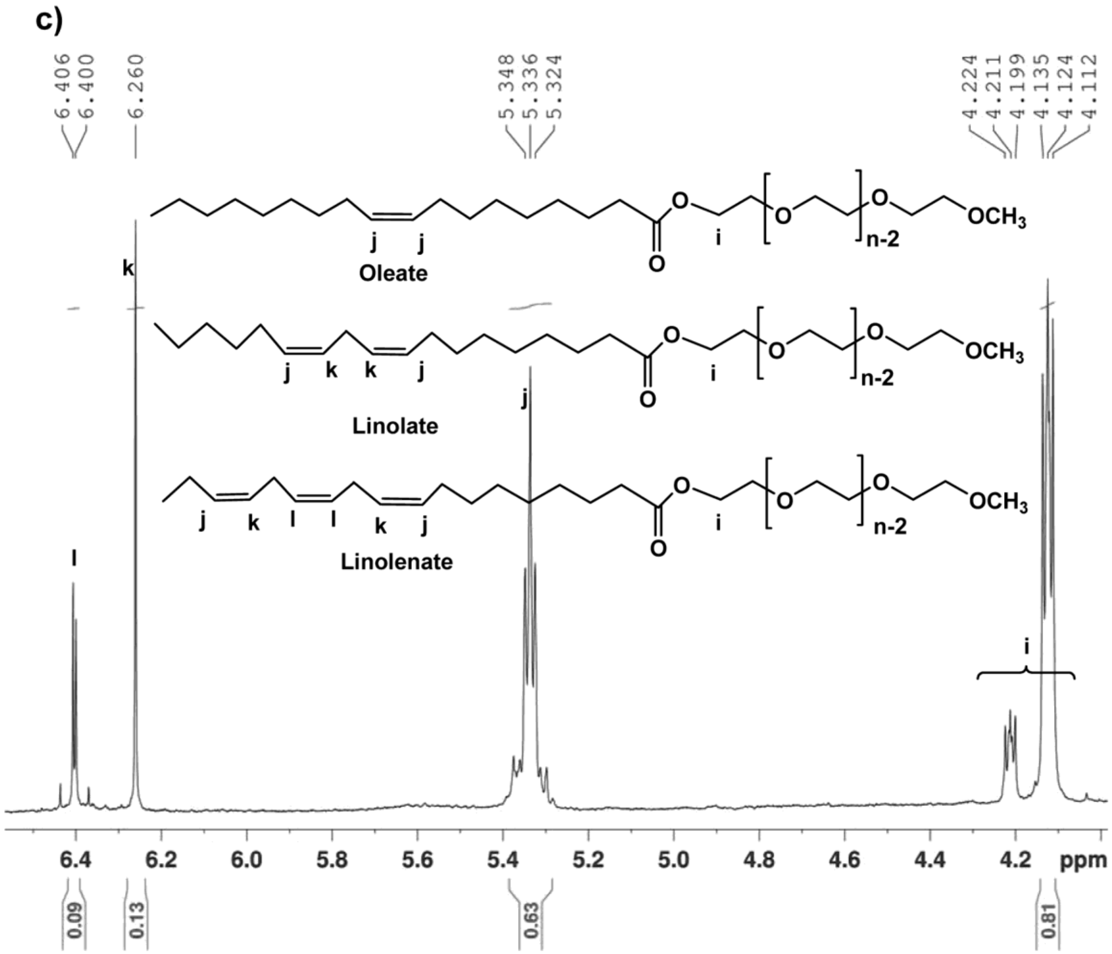

Supplemental data Fig. 4.

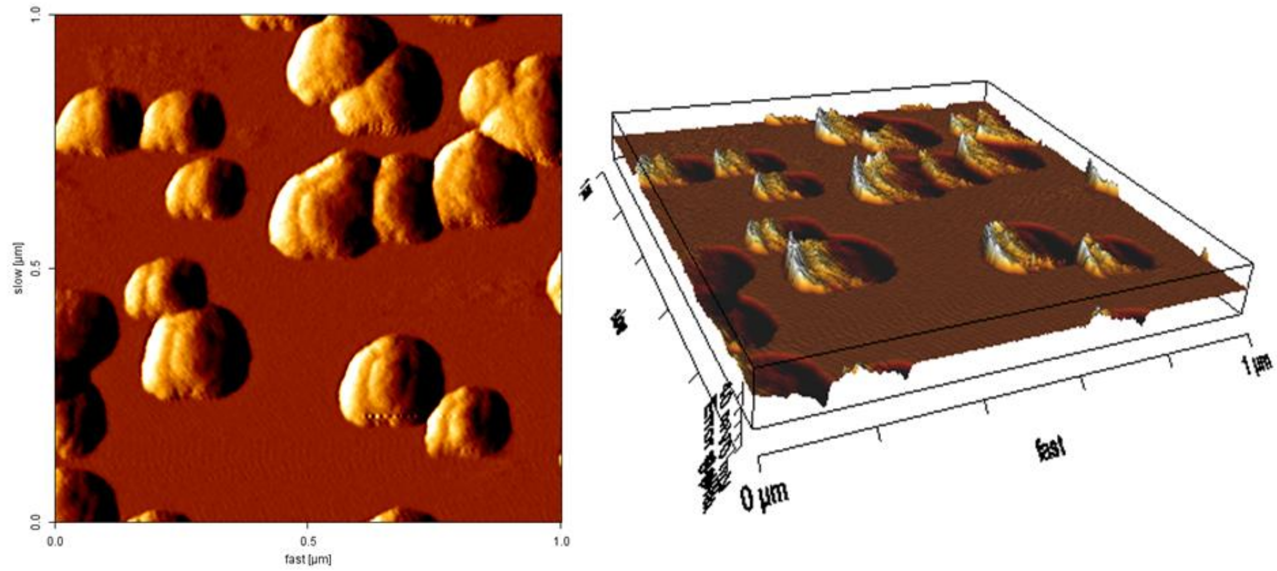

Supplemental data Fig. 5.

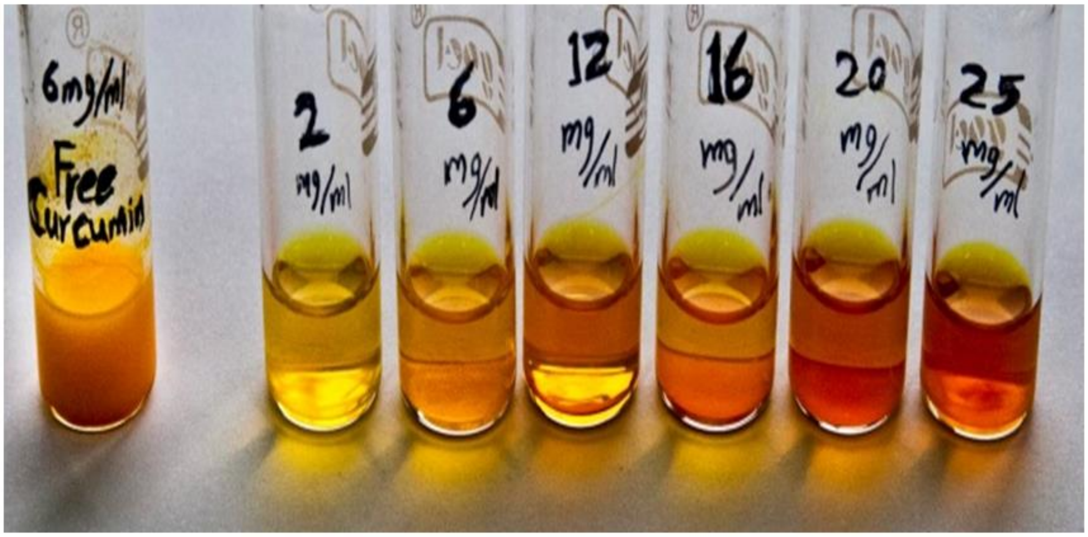

Supplemental data Fig. 6.

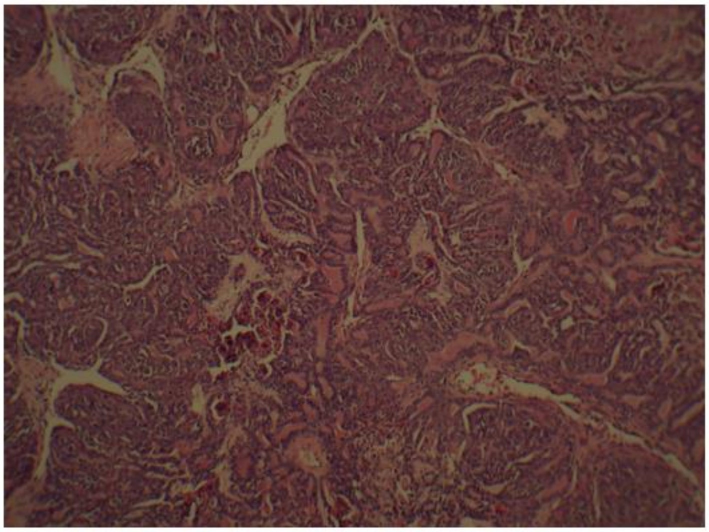

Supplemental data Fig. 7.

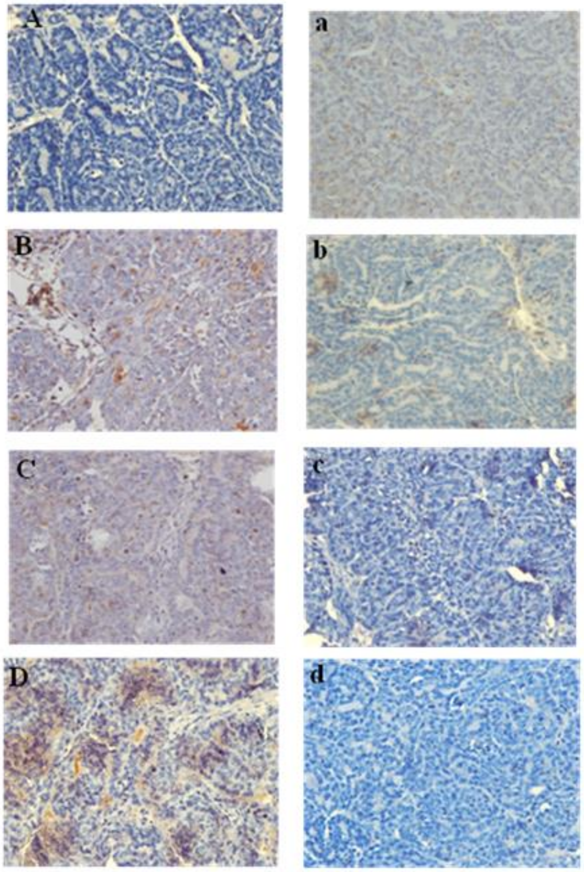

Supplement: Supplementary file 1 — This content was submitted by the authors as supplemental material for an original article published in BioMed Research International. The content is presented as the author submitted it. All questions regarding the supplemental data should be directed to the corresponding author of the published article. It provides more experimental results on the encapsulation of curcumin in diblock copolymer micelles for cancer therapy. [file 824746.f1.pdf]
